# Shockwave Lithotripsy Versus Ureteroscopic Treatment as Therapeutic Interventions for Stones of the Ureter (TISU): A Multicentre Randomised Controlled Non-inferiority Trial^☆^

**DOI:** 10.1016/j.eururo.2021.02.044

**Published:** 2021-07

**Authors:** Ranan Dasgupta, Sarah Cameron, Lorna Aucott, Graeme MacLennan, Ruth E. Thomas, Mary M. Kilonzo, Thomas B.L. Lam, James N’Dow, John Norrie, Ken Anson, Neil Burgess, Charles T. Clark, Francis X. Keeley, Sara J. MacLennan, Kath Starr, Sam McClinton

**Affiliations:** a Department of Urology, Imperial College Healthcare NHS Trust, London, W2 1NY, UK; b Centre for Healthcare Randomised Trials, University of Aberdeen, Health Sciences Building, Foresterhill, Aberdeen, UK; c Health Services Research Unit, University of Aberdeen, Health Sciences Building, Foresterhill, Aberdeen, UK; d Health Economics Research Unit, University of Aberdeen, Aberdeen, UK; e NHS Grampian, Department of Urology, Aberdeen Royal Infirmary, Aberdeen, UK; f Edinburgh Clinical Trials Unit, Usher Institute of Population Health Sciences & Informatics, University of Edinburgh, Edinburgh, UK; g Department of Urology, St Georges University Hospitals NHS Foundation Trust, London, UK; h Department of Urology, Norfolk and Norwich University Hospitals NHS Foundation Trust, Norwich, UK; i Stone Patient Advisory Group, Section of Endourology, British Association of Urological Surgeons, London, UK; j Bristol Urological Institute, North Bristol NHS Trust, Bristol, UK; k Academic Urology Unit, University of Aberdeen, Health Sciences Building, Foresterhill, Aberdeen, UK; l Nottingham Clinical Trials Unit, University of Nottingham, University Park, Nottingham, UK

**Keywords:** Therapeutic interventions for symptomatic ureteric stones, Ureteric stones, Ureteroscopy, Extracorporeal shockwave lithotripsy, Trial, Randomised controlled trial

## Abstract

**Background:**

Renal stone disease is common and can cause emergency presentation with acute pain due to ureteric colic. International guidelines have stated the need for a multicentre randomised controlled trial (RCT) to determine whether a non-invasive outpatient (shockwave lithotripsy [SWL]) or surgical (ureteroscopy [URS]) intervention should be the first-line treatment for those needing active intervention. This has implications for shaping clinical pathways.

**Objective:**

To report a pragmatic multicentre non-inferiority RCT comparing SWL with URS.

**Design, setting, and participants:**

This trial tested for non-inferiority of up to two sessions of SWL compared with URS as initial treatment for ureteric stones requiring intervention.

**Outcome measurements and statistical analysis:**

The primary outcome was whether further intervention was required to clear the stone, and secondary outcomes included quality of life assessment, severity of pain, and serious complications; these were based on questionnaires at baseline, 8 wk, and 6 mo. We included patients over 16 yr with a single ureteric stone clinically deemed to require intervention. Intention-to-treat and per-protocol analyses were planned.

**Results and limitations:**

The study recruited between July 1, 2013 and June 30, 2017. We recruited 613 participants from a total of 1291 eligible patients, randomising 306 to SWL and 307 to URS. Sixty-seven patients (22.1%) in the SWL arm needed further treatment compared with 31 patients (10.3%) in the URS arm. The absolute risk difference was 11.7% (95% confidence interval 5.6%, 17.8%) in favour of URS, which was inside the 20% threshold we set for demonstrating noninferiority of SWL.

**Conclusions:**

This RCT was designed to test whether SWL is non-inferior to URS and confirmed this; although SWL is an outpatient noninvasive treatment with potential advantages both for patients and for reducing the use of inpatient health care resources, the trial showed a benefit in overall clinical outcomes with URS compared with SWL, reflecting contemporary practice. The Therapeutic Interventions for Stones of the Ureter (TISU) study provides new evidence to help guide the choice of modality for this common health condition.

**Patient summary:**

We present the largest trial comparing ureteroscopy versus extracorporeal shockwave lithotripsy for ureteric stones. While ureteroscopy had marginally improved outcome in terms of stone clearance, as expected, shockwave lithotripsy had better results in terms of health care costs. These results should enable patients and health care providers to optimise treatment pathways for this common urological condition.

## Take Home Message

This level 1 evidence highlights only a slight difference between ureteroscopy (URS) and shockwave lithotripsy (SWL), supporting SWL as an outpatient treatment without anaesthetic or theatre requirements, particularly relevant after COVID-19. While URS had higher stone clearance, both modalities appear suitable for primary treatment choice.

## 1 Introduction

The rising incidence of renal stone disease is well documented globally, with concomitant increased pressure on health care resources and in particular for the emergency management of acute ureteric colic [1]. While there has been a recent focus on conservative medical management (medical expulsive therapy) [2], the greater challenge is how to manage stones that do not pass spontaneously and need active intervention. These patients are often multiple attenders at emergency departments, frequently require admission to an inpatient ward, and ultimately may need active surgical management.

In the emergency setting, a kidney obstructed by a stone can be drained with a temporary nephrostomy or ureteric stent, while the definitive surgical management to fragment the stone involves either shockwave lithotripsy (SWL) or laser fragmentation with ureteroscopy (URS). The delivery of SWL can be in an outpatient setting (by the use of either a fixed-site or a mobile lithotripter), whereas URS typically requires general anaesthesia and is undertaken in an operating theatre. Despite the availability of both modalities for over 2 decades, the choice of modality is usually based on local resources and expertise, patient choice, and accessibility. This is due to the lack of high-quality evidence on which both patients and clinicians can base treatment choices [3].

We describe and report the results of a large pragmatic randomised multicentre study comparing SWL with URS (Therapeutic Interventions for symptomatic Stones of the Ureter Stones of or TISU study) assessing clinical outcomes of importance to patients (ie, need for further intervention) and important secondary outcome measures such as quality of life, in order to provide guidance to patients, clinicians, and health care commissioners.

## 2 Patients and methods

### 2.1 Study design and participants

In this randomised, non-inferiority trial, we recruited patients with a diagnosis of a unilateral ureteric stone from 25 UK National Health Service (NHS) hospitals. A prerequisite for involvement was that the site had a “fixed-site” lithotripter. We included adults aged ≥16 yr with stones of any size, confirmed by noncontrast computed tomography of the kidneys, ureters, and bladder (CTKUB) and deemed to require intervention, and satisfying the following inclusion and exclusion criteria:

The inclusion criteria were as follows:

1 Presence of stone confirmed by CTKUB (including a scout film)
2 Presence of a ureteric stone requiring intervention (either as a primary intervention or after failed conservative management)
3 Age ≥16 yr
4 Single ureteric stone of any size requiring treatment
5 Clinical suitability of patients for either SWL or URS treatment
6 Ability of patients to give written informed consent, which includes adherence to the requirements of the trial

The exclusion criteria were as follows:

1 Pregnancy
2 Stones not confirmed by CTKUB
3 Bilateral ureteric stones
4 Abnormal urinary tract anatomy (such as horseshoe kidney or ileal conduit)
5 Inability of patients to understand or complete trial documentation

We provided patients with a TISU patient information sheet, and if they decided to take part they were asked to provide written informed consent. Trained site personnel, research nurses, and clinicians enrolled participants to the trial.

The trial was approved by the North of Scotland Ethics Committee 1 (reference: 13/NS/0002), by the sponsors NHS Grampian and the University of Aberdeen, and by the R&D departments of the NHS organisations at each participating site. We conducted the trial in accordance with the principles of Good Clinical Practice and registered it on the UK Clinical Research Network Portfolio (UKCRN study ID 13979), and were assigned an International Standard Randomised Clinical Trial number (ISRCTN92289221). Full details of the methods are available in the published protocol [4].

### 2.2 Randomisation

Participants were allocated in a 1:1 ratio to either SWL or URS by a centralised randomisation system hosted at the Centre for Healthcare Randomised Trials (CHaRT) in Aberdeen. The minimisation covariates were centre, stone size (≤10 or >10 mm), and stone location (upper, middle, or lower ureter).

### 2.3 Procedures

We collected baseline clinical data and participant-reported quality of life and pain before randomisation. Following randomisation, patients were provided with a date for their allocated intervention. We asked participants to complete follow-up questionnaires at 8 wk and 6 mo after randomisation. Site staff provided clinical information via case report forms at these same time points.

The principal investigator at each site was responsible for grading and reporting suspected serious adverse events to the trial office for the chief investigator to confirm. The sponsor and research ethics committee monitored all safety events. We did not collect non-serious adverse events.

### 2.4 Outcomes

The primary clinical outcome was the resolution of stone episode, defined as no further intervention required to facilitate stone clearance up to 6 mo from randomisation. However, when sharing results with collaborators, it became clear that results were easier to interpret in the non-inferiority framework using the complement of this binary outcome, that is, the need for further treatment, so we have expressed the results as such. All further interventions received (and any serious complications [Clavien-Dindo level ≥3]) were collected at 8 wk and 6 mo after randomisation. The secondary outcomes were pain (numerical rating scale), self-reported number of days of analgesic use, quality of life using EQ-5D-3L [5] (using the UK value sets), and the Short Form (SF)-12 physical (PCS) and mental (MCS) component summary scores [6].

### 2.5 Statistical analysis

Our sample size calculations assumed, based upon evidence from the most recent systematic review, that the stone-free without further intervention proportions were 0.75 (P1) in the URS arm and 0.65 (P2) in the SWL arm [3]. We used a noninferiority margin of 20% (so P2 – P1> –0.20 to infer noninferiority). Simulation of thousands of trials of fixed sizes with the parameters P1 and P2 as above indicated that a trial of 450 per arm was required for the lower bound of the estimated 95% confidence interval (CI) to exclude –20% with 90% power. We increased this to 1000 for attrition to the primary outcome (due to potential for crossover) as our planned analysis approach was a per-protocol one (ie, excluding crossovers). The non-inferiority margin came from a survey of members of the British Association of Urological Surgeons Section of Endourology. Following slower than planned recruitment, our funders requested a reassessment of the assumptions behind the sample size calculation. This was done by discussing the trial-aggregated primary outcome from 267 participants with our independent Data Monitoring Committee (DMC) and subsequently with the funders, and the sample size was revised from 1000 to 750. The view of the DMC and funders was that a per-protocol analysis was of secondary importance to the intention-to-treat (ITT) approach. Therefore, using all available data for the primary analysis (and with the original design of assuming that the stone-free proportions would be 65% in the SWL arm and 75% in the URS arm), the achievable sample size of 750 gave above 80% power (full details in the Supplementary material).

Treatment groups are described at baseline and follow-up using means (with standard deviations), medians (with interquartile ranges), and numbers (with percentages) where relevant. We analysed the primary outcome using a generalised linear model with a log-link function and robust error variance, to estimate covariate-adjusted relative risks and derive risk differences [7]. Models were adjusted for the design covariates of trial centre (random effect), stone size (≤10 and >10 mm), stone location (upper, middle, or lower ureter), and age and gender. Our main approach to analysing the primary outcome was ITT given the pragmatic nature of TISU evaluating two care pathways. In the analysis ITT-1, all participants were included “as randomised”, including those passing their stone before their intervention (this reflects waiting times for both SWL and URS in the UK National Health Service) setting. A second analysis, labelled ITT-2, repeated this but excluded all participants who passed their stones before the first randomised intervention. An ITT approach can be conservative for a non-inferiority trial, so we also prespecified per-protocol analyses. Results labelled PP-1 and PP-2 mirror the ITT analyses above but included only participants who were treated in line with the care pathway they were allocated to (ie, excluding crossovers). The primary outcome reflects the number requiring further intervention; thus, a higher figure is a worse outcome. Consequently, in order to avoid double negatives, we used the upper bound of the confidence interval around the absolute risk difference (estimated from our models) ruling out the prespecified noninferiority margin of 20% to conclude noninferiority. We made no adjustment for missing data because we had complete outcome data (apart from six postrandomisation exclusions) on all participants who gave consent for their clinical data to be used.

Secondary outcomes were compared in a similar way using a generalised linear model appropriate for the distributional form of the outcome being analysed, but in a superiority framework. We used linear mixed models for repeated measures quality of life data, estimating treatment effects by including a time-by-treatment interaction for fixed (nominal) time points of 8 wk and 6 mo from randomisation. We used a multiple imputation approach to deal with missing EQ-5D-3L and SF-12 outcome data. For each of these outcomes, we generated 50 imputation sets for each arm of the trial separately. Our imputation model used the baseline and 8-wk scores, treatment received, stone size, stone location (upper, middle, and lower ureter), gender, age, centre, and primary outcome status to predict missing EQ-5D-3L and SF-12 scores.

We explored the moderating effect of three a priori subgroup variables on the primary outcome by including subgroup-by-treatment interactions in our primary outcome model. These were stone size (≤10 and >10 mm), stone location (upper, middle, or lower ureter), and gender. We used Forest plots to summarise the within-subgroup treatment estimates using 99% CIs. We used Stata 15 (StataCorp LP, College Station, TX, USA) for all our statistical analyses [8].

### 2.6 Role of funding source

The funder approved the study proposal but had no role in the collection, analysis, or interpretation of data, or writing of the report. The senior author had full access to all the data in the study and had final responsibility for the decision to submit for publication.

## 3 Results

**Fig. 1:**
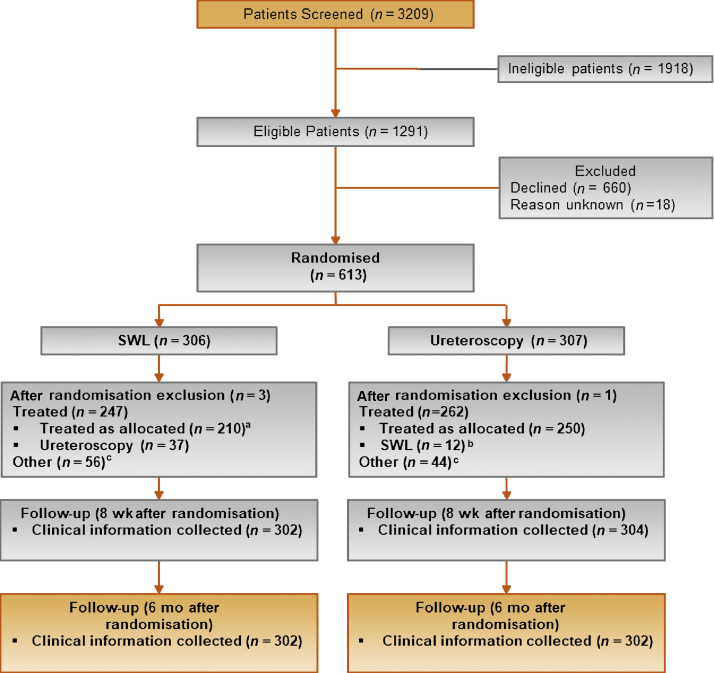
Consort diagram. SWL = shockwave lithotripsy; URS = ureteroscopy. ^a^ Eighty-six patients had two sessions of SWL. ^b^ SWL arm: other = stone passed before treatment (*n* = 52) and not treated within the NHS/did not attend/unable to treat (*n* = 4); URS arm: other = stone passed before treatment (*n* = 36), and not treated within the NHS/did not attend/unable to treat (*n* = 8). ^c^ Three had two sessions of SWL.

Between July 1, 2013 and June 30, 2017, we randomised 613 (19% of 3209 screened) participants from 25 UK secondary care centres. A total of 306 were allocated to SWL and 307 to URS; the groups were well balanced at baseline (Table 1). In the SWL arm, 37 (12%) patients did not receive allocated treatment and had URS (reasons for change from allocated treatment included medical reasons, stone not seen at the time of SWL, and patient choice). In the URS arm, 12 (4%) did not receive their allocated treatment and received SWL (reasons for change being medical reasons and patient choice). For 52 (17%) in the SWL arm and 36 (12%) in the URS arm, their stone passed prior to treatment (ie, pretreatment imaging showed no stone visible and symptoms had resolved). Participant flow through the trial is summarised in a CONSORT diagram (Fig. 1).

**Table 1:** Baseline characteristics

Variable	SWL	URS
	*N* = 303	*N* = 306
Age (yr)	52.1 (41.6, 61.7)	50.0 (39.5, 60.3)
Male	241 (80)	234 (80)
Ureteric stone size (mm)	6.0 (5.0, 8.0)	6.0 (5.0, 8.0)
Ureteric stone size ≤10 mm	288 (95)	292 (95)
Stone location		
Upper ureter	138 (46)	139 (45)
Middle ureter	47 (16)	50 (16)
Lower ureter	118 (39)	117 (38)
Currently taking analgesic medications		
Yes	220 (73)	193 (63)
No	64 (21)	96 (31)
Missing	19 (6)	17 (6)
Level of pain today	**301:** 3.0 (1.0, 6.0)	**303:** 3.0 (1.0, 6.0)
Pain related to ureteric stone during last 7 d		
Had pain	236 (78)	232 (76)
No pain	63 (21)	69 (23)
Missing	4 (1)	5 (2)
Number of patients taking pain medication during last 7 d	**298:** 4 (2, 7)	**300:** 3.0 (1, 6)
EQ-5D	**298:** 0.796 (0.691, 1)	**297:** 0.796 (0.689, 1)
EQ-5D visual scale	**283:** 75.0 (50.0, 87.0)	**284:** 75.0 (50.0, 90.0)
SF-12 PCS	**290:** 44.1 (38.1, 50.5)	**289:** 46.1 (38.2, 52.9)
SF-12 MCS	**290:** 49.7 (40.9, 57.7)	**289:** 52.2 (44.1, 57.6)

SWL = shockwave lithotripsy; SF-12 MCS = Short Form-12 mental component summary score; SF-12 PCS = Short Form-12 physical component summary score; URS = ureteroscopy.

Cells are *n* (%) or *n*: median (quartile 1, quartile 3).

In our primary analysis (ITT-1), there were 67 patients (22.1%) in the SWL arm who needed further treatment and 31 patients (10.3%) in the URS arm (Table 2). The ITT-1 results give the absolute risk difference as 11.7% (95% CI 5.6–17.8), with the upper bound of the 95% CI inside the threshold for noninferiority. When excluding the participants who passed their stone before treatment (ITT-2, PP-2), the estimates favoured URS more, so that, for the per-protocol analysis (PP-2), SWL can no longer be considered noninferior to URS. Results are summarised visually in Supplementary Figure 1. Within-subgroup treatment effects are summarised in Supplementary Figure 2 and were fairly homogeneous across all strata; there was no evidence that subgroup moderated treatment effects.

**Table 2:** Primary outcome: proportion requiring further intervention to clear the stone for SWL compared with URS

Population	SWL		URS		ARD a, b	95% CI	Non-inf *p* value	RR a, b	95% CI
	*n*/*N*	%	*n*/*N*	%					
ITT-1	67/302	22	31/302	10	0.12	(0.06, 0.18)	0.004	2.13	(1.37, 3.32)
ITT-2	65/250	26	31/266	12	0.14	(0.07, 0.21)	0.051	2.19	(1.41, 3.40)
PP-1	64/262	24	27/283	10	0.15	(0.08, 0.21)	0.046	2.52	(1.60, 3.94)
PP-2	62/210	30	27/247	11	0.18	(0.10, 0.26)	0.31	2.63	(1.67, 4.15)

ARD = absolute risk difference (SWL – URS); CI = confidence interval; ITT-1 = intention-to-treat analysis including all participants; ITT-2 = intention-to-treat analysis excluding those who passed their stone prior to any intervention; PP-1 = per-protocol analysis including those who passed their stone before treatment; PP-2 = per-protocol analysis excluding those who passed their stone before treatment; RR = relative risk (URS is the reference category); SWL = shockwave lithotripsy; URS = ureteroscopy.

Non-inf *p* value indicates noninferiority *p* value for the ARD results only: SWL is inferior to URS.

a All treatment effect estimates adjusted for outcome at baseline, stone size, stone location, age, gender, and centre.

b Modified Poisson regression model with a log-link function and robust error variance.

The number of treatment-related complications was similar in both care pathways (Table 3). The frequency of serious complications was similar in each arm (3.6% [9/247] in the SWL arm and 2.7% [7/262] in the URS arm). There was only one death, which occurred in the URS group and was unrelated to the treatment; this low number (<1%) is as expected, as these procedures are generally associated with extremely low mortality figures. There were two life-threatening complications, both in the URS group—one was cardiac (in a patient with a previous myocardial infarction) and the other pulmonary (pulmonary embolus, in a patient who had also undergone recent orthopaedic surgery); neither was assessed as attributable to the trial intervention (or anaesthesia). There were slightly fewer complications (in participants who received any treatment) in the URS pathway than in the SWL pathway, but with few events there was considerable uncertainty around the differences.

**Table 3:** Treatment-related complications (secondary) up to 6 mo after randomisation by allocated and received treatment: comparing SWL with URS (reference)

Participants with treatment-related complication	SWL a	URS b	ARD c, d	95% CI	RR c, d	95% CI
*n* (%)	9/244 (4%)	7/256 (3%)	0.01	(–0.02, 0.04)	1.34	(0.47, 3.80)

	Received SWL	Received URS				
*n* (%)	7/221 (3%)	9/279 (3%)	0.00	(–0.04, 0.03)	0.93	(0.31, 2.81)

ARD = absolute risk difference (SWL – URS); CI = confidence interval; RR = relative risk (URS is the reference category); SWL = shockwave lithotripsy; URS = ureteroscopy.

Denominators are those participants who received any treatment.

a Excluding one who withdrew consent for use of their 6-mo clinical data.

b Excluding one who died before 6 mo and three who withdrew consent for use of their 6 mo clinical data.

c All treatment effect estimates adjusted for outcome at baseline, stone size, stone location, age, gender, and centre.

d Modified Poisson regression model with a log-link function and robust error variance.

**Fig. 2:**
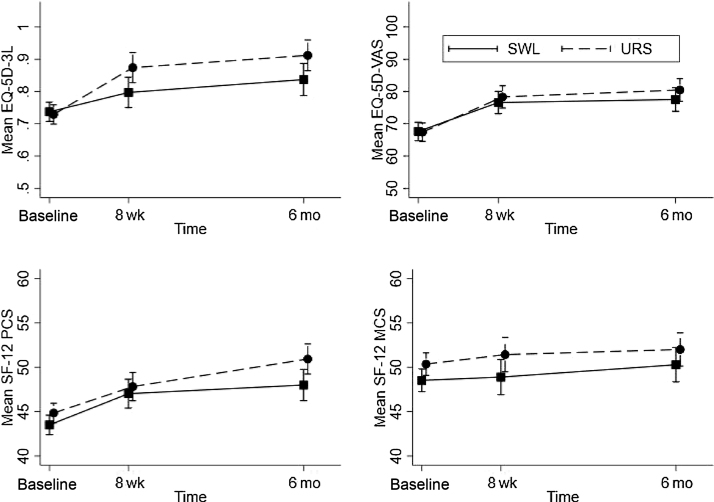
Plots over time of complete case quality of life data using EQ-5D-3L (mean score and VAS) and the SF-12 physical (PCS) and mental (MCS) component scores. EQ-5D-3L is a complex composite of five questions scoring 0–2, where 0 represents “no problem”. Ultimately, these are combined such that the maximum score is 1, indicating the best health state, using the UK value set. EQ-5D VAS is a visual analogue scale ranging from 0 to 100, whereby 100 indicates the best health status. SF-12v2: Physical and mental health composite scores (PCS and MCS) are scores from 12 questions ranging from 0 to 100; for each scale, 0 indicates the lowest level of health measured and 100 indicates the highest level of health. SF-12 = Short Form-12; SWL = shockwave lithotripsy; URS = ureteroscopy; VAS = visual analogue scale.

Secondary outcomes are reported in Table 4. We found a similar pattern between care pathways for both the pain measures and the acceptability measure. At 8 wk, self-reported pain was low. The participants in the SWL arm reported taking pain relievers more frequently (incidence rate ratio 1.38 [0.93, 2.06]), but the number of days of taking pain relievers was small in both groups. Of those who responded, over 80% in each arm stated that they would recommend their treatment to a friend with ureteric stones, and there was no evidence that this differed between arms. Quality of life, as measured by EQ-5D-3L (the mean and visual scale) and the SF-12 PCS and MCS components are reported in Table 4 and Figure 2. The trial quality of life improved over the duration of the trial from baseline to 6 mo in both arms. Nonresponders tended to be male and younger in age. When we used observed data only, there were small but consistent effects favouring URS for both the SF-12 PCS and MCS and the EQ-5D-3L visual analogue scale with potentially more sustained improvements from 8 wk for the EQ-5D-3L score. However, these effects were attenuated when we used multiple imputation models. Waiting times in days are described in Table 5. Over 90% of participants in the SWL care pathway received treatment within 8 wk, and a slightly lower proportion (86%) of participants in the URS care pathway were treated within 8 wk (excluding those who passed their stone before treatment in both pathways).

**Table 4:** Other secondary outcomes: comparison of SWL with URS (reference)

Outcome	SWL	URS	Effect size a	95% CI	Imputed Effect b	Imputed (95% CI) b
Pain today, c*n*	183	184				
Median (IQR)	0 (0, 2)	0 (0, 1)				
Mean (SD)	1.3 (2.4)	0.97 (2.0)	0.3 d	(–0.2, 0.9)		
Days of pain relief (last 7 d), c*n*	178	181				
Median (IQR)	0 (0, 2)	0 (0, 1)				
Mean (SD)	1.5 (2.5)	1.0 (1.9)	1.38 e	(0.9, 2.1)		
Recommend to a friend, c*n*	171	171				
x (%)	148 (87%)	142 (83%)	1.04 f	(1.0, 1.1)		
EQ-5D-3L g (over time)						
Baseline, *n*	298	297				
Mean (SD)	0.737 (0.263)	0.729 (0.303)				
8 wk, *n*	149	152				
Mean (SD)	0.797 (0.260)	0.874 (0.207)	–0.080 h	(–0.145, –0.015)	–0.1	(–0.1, –0.0)
6 mo, *n*	130	143				
Mean (SD)	0.837 (0.289)	0.912 (0.182)	–0.086 h	(–0.148, –0.024)	–0.1	(–0.1, 0.0)
EQ-5D VAS i (over time)						
Baseline, *n*	282	283				
Mean (SD)	67.6 (24.5)	67.4 (26.5)				
8 wk, *n*	148	149				
Mean (SD)	76.6 (21.5)	78.3 (20.7)	–4.5 h	(–8.7, –0.3)	–3.0	(–8.0, 1.9)
6 mo, *n*	129	138				
Mean (SD)	77.5 (21.2)	80.5 (17.9)	–3.5 h	(–8.0, 0.9)	–2.6	(–7.9, 2.6)
SF-12 PCS j (over time)						
Baseline, *n*	290	289				
Mean (SD)	43.5 (9.5)	44.8 (9.7)				
8 wk, *n*	150	156				
Mean (SD)	47.0 (10.1)	47.9 (9.2)	–0.1 h	(–2.0, 1.9)	–0.9	(–2.4, 0.6)
6 mo, *n*	137	146				
Mean (SD)	48.0 (10.5)	50.9 (8.8)	–2.0 h	(–4.0, –0.0)	–1.7	(–3.2, –0.2)
SF-12 MCS j (over time)						
Baseline, *n*	290	289				
Mean (SD)	48.5 (11.1)	50.4 (9.6)				
8 wk, *n*						
Mean (SD)	48.9 (12.4)	51.4 (9.9)	–2.2 h	(–4.5, –0.0)	–1.2	(–3.0, 0.5)
6 mo, *n*	137	146				
Mean (SD)	50.3 (11.6)	52.0 (10.4)	–1.1 h	(–3.4, 1.2)	–1.3	(–3.0, 0.5)

CI = confidence interval; IQR = interquartile range; SF-12 MCS = Short Form-12 mental component summary score; SF-12 PCS = Short Form-12 physical component summary score; SD = standard deviation; SWL = shockwave lithotripsy; URS = ureteroscopy.

a All treatment effect estimates adjusted for outcome at baseline (where relevant), stone size, stone location, age, gender, and centre.

b Multiple imputation process included the primary outcome and was conducted on each treatment group separately. It also included all time points of the outcome.

c At 8 wk.

d Mean difference between SWL and URS.

e Incident rate ratio from negative binomial regression model.

f Relative risk.

g EQ-5D-3L 1 indicates “best health state”.

h Linear mixed model for repeated time points.

i EQ VAS (visual analogue scale) ranges from 0 (“worst imaginable health state”) to 100 (“best imaginable health state”).

j SF-12v2; the physical score (PCS) and a mental score (MCS) are standardised to have a mean of 50 and an SD of 10, where 100 indicates the highest level of health.

**Table 5:** Waiting time in days from randomisation to treatment

Treatment allocation	*N*	Median (IQR)	Range
SWL (303)			
SWL pathway: treated	247	8 (2–18)	(0–415)
Treated as randomised	210	7 (2–15)	(0–79)
Switched treatment	37	25 (2–70)	(0–415)
Proportion treated within 8 wk	229/247	(93%)	
URS (306)	.		
URS pathway: treated	261	25 (9–44)	(0–269)
Treated as randomised	250	25 (9–44)	(0–269)
Switched treatment	12	22 (2–47)	(0–84)
Proportion treated within 8 wk	225/261	(86%)	

IQR = interquartile range; SWL = shockwave lithotripsy; URS = ureteroscopy.

## 4 Discussion

In our trial, we found that the further intervention rate at 6 mo in the SWL arm was 11.7% (95% CI 5.6–17.8%) higher than that in the URS arm, but this difference was non-inferior. That is, it was within the 20% limit set at the outset of the study to demonstrate non-inferiority, which would make it acceptable to patients and urologists as the initial treatment pathway. However, our results, as demonstrated by the per-protocol analysis on those who did not clear or pass their stone prior to treatment, corroborate previous findings in demonstrating better stone clearance with URS [3], [9].

Once the clinical decision to pursue active intervention is made, two main pathways are available: treatment that starts with SWL and treatment that starts with URS. There was uncertainty about the balance between clinical effectiveness and economic effectiveness when comparing the two treatments and TISU was designed to address that. This uncertainty reflects the differences in delivery of the two treatments, the clinical outcomes achieved, and the costs associated with each. It also reflects the fact that, for patients, there are major differences between the treatments in terms of waiting times for treatment, invasiveness, need for general anaesthesia, time in hospital, and time to full recovery.

A key factor in decision-making between these treatment pathways for both patients and clinicians is complications. For the purposes of this study, we did not collect data on the expected low-grade complications of these treatments, which have been well reported [10], but focussed on serious complications, and these were very similar for SWL (4%) and URS (3%) pathways.

We assessed quality of life using two generic quality of life measures (EQ-5D-3L and SF-12). With these generic quality of life measures (not specific to this condition), there was improvement from baseline for both the URS and the SWL arms, which was maintained for up to 6 mo. We acknowledge the potential limitation in this regard. Firstly, the response rate to questionnaires was low at both follow-up time points, although this is not uncommon for ureteric stones trials. Estimates on observed data favoured URS, but these were attenuated when we accounted for missing data. However, CIs around imputed estimates rule out any clinically meaningful differences in quality of life. Secondly, given no condition-specific quality of life tool for ureteric stones, generic quality of life tools may be too blunt to pick up differences between care pathways at the earlier time point.

Blinding was not possible given the nature of the interventions in each care pathway. We do not consider this a weakness; rather it reflects the pragmatic nature of our trial, embedded in current urological practice across the UK. Our protocol allowed for “crossover” as both treatments are available and changes in the clinical condition of participants may dictate a change of approach, and indeed, as anticipated, this occurred. This reflects real-life clinical decision-making in symptomatic patients, and it is unlikely that this was affected by participants being recruited in the trial. Other factors may influence patient choice (as seen in the crossover patients, eg, need for anaesthesia) and clinician choice such as health care resource usage (access to operating theatre and beds). There were also a proportion of participants who passed their stones before treatment could occur, reflecting the real-life waiting times for these procedures. However, both ITT and per-protocol analyses—with and without participants who received either their allocated SWL or their allocated URS treatment—showed broadly consistent results. Our results on the comparative effectiveness of the URS and SWL pathways are generalisable to other settings because these two pathways are widely available and utilised in urological practice worldwide.

## 5 Conclusions

Our primary analysis found that SWL can be considered non-inferior to URS. The broader picture over a range of sensitivity analyses is that URS is consistently better than SWL in terms of the need for further intervention, but that procedures can be included in care pathways, with decisions about which to use based on local stakeholders’ circumstances and preferences. The evidence from TISU provides a firm basis to inform decisions, at either individual patient level or health care commissioning level in general.

  ***Author contributions*:** Ranan Dasgupta had full access to all the data in the study and takes responsibility for the integrity of the data and the accuracy of the data analysis.

  *Study concept and design*: McClinton, Keeley, Anson, N'Dow.

*Acquisition of data*: Dasgupta, Cameron, Starr, McClinton, Anson, Keeley, Thomas.

*Analysis and interpretation of data*: McClinton, Dasgupta, N'Dow, Anson, Keeley, Cameron, Starr, G. MacLennan, S. MacLennan, Aucott, Norrie, Thomas.

*Drafting of the manuscript*: McClinton, Dasgupta, Cameron, Starr, G. MacLennan, S. MacLennan, Aucott, Norrie, Thomas.

*Critical revision of the manuscript for important intellectual content*: McClinton, Dasgupta, N'Dow, Anson, Keeley, Cameron, Starr, G. MacLennan, S. MacLennan, Aucott, Norrie, Thomas.

*Statistical analysis*: G. MacLennan, S. MacLennan, Aucott, Norrie.

*Obtaining funding*: McClinton, N'Dow, Anson, Keeley, Cameron.

*Administrative, technical, or material support:* Cameron, Starr, Thomas.

*Supervision*: McClinton, Anson, N'Dow.

*Other*: None.

  ***Financial disclosures:*** Ranan Dasgupta certifies that all conflicts of interest, including specific financial interests and relationships and affiliations relevant to the subject matter or materials discussed in the manuscript (eg, employment/affiliation, grants or funding, consultancies, honoraria, stock ownership or options, expert testimony, royalties, or patents filed, received, or pending), are the following: Professor N'Dow was a member of the HTA General Committee (2016–2019). Dr. Aucott is a member of PHR Research Funding Board (2017–2021). Professor John Norrie reports grants from University of Aberdeen and University of Edinburgh, during the conduct of the study, and declares membership of the following NIHR boards: CPR Decision-making Committee, HTA Commissioning Board, HTA Commissioning Sub-Board (EOI), HTA Funding Boards Policy Group, HTA General Board, HTA Post-Board funding teleconference, NIHR CTU Standing Advisory Committee, NIHR HTA & EME Editorial Board, and Pre-exposure Prophylaxis Impact Review Panel. Mr. Keeley is a member of a committee assisting with the development of a new ureteroscope (Boston Scientific) and of the advisory committee of Olympus Medical. All other authors declare no competing interests.

  ***Funding/Support and role of the sponsor*:** This paper presents independent research funded by the National Institute for Health Research (NIHR) Health Technology Assessment (HTA) Programme, Project Number 10/137/01. The views and opinions expressed in this report are those of the authors and do not necessarily reflect those of the HTA Programme, the NIHR, the NHS, or the Department of Health. The Health Services Research Unit is core funded by the Chief Scientist Office of the Scottish Government Health and Social Care Directorates; however, the opinions expressed in this publication are those of the authors and might not be shared by the Chief Scientist Office.

  ***Acknowledgements*:** Special thanks must go to all of the TISU trial participants and staff at each of our recruiting centres for taking part in this trial. We thank our collaborators at the TISU sites, and participating National Health Service (NHS) trusts and boards (the Newcastle upon Tyne Hospitals NHS Foundation Trust, South Tees Hospitals NHS Foundation Trust, City Hospitals Sunderland NHS Foundation Trust, the Leeds Teaching Hospitals NHS Trust, University Hospitals Bristol NHS Trust, NHS Lothian, Guy’s and St Thomas’ NHS Foundation Trust, St George’s Healthcare NHS Trust, Mid Yorkshire Hospitals NHS Trust, Sheffield Teaching Hospitals NHS Foundation Trust, Cambridge University Hospitals NHS Foundation Trust, Central Manchester University Hospitals NHS Foundation Trust, Betsi Cadwaladr University Health Board, Dartford and Gravesham NHS Trust, St Helens & Knowsley Teaching Hospital NHS Trust, Ashford & St Peters Hospitals NHS Foundation Trust, Imperial College Healthcare NHS Trust, Northwest London Hospital NHS Trust, Mid-Essex Hospital NHS Trust, Salford Royal NHS Foundation Trust, Oxford University Hospitals Trust, East Kent Hospitals University NHS Foundation Trust, Hull and East Yorkshire Hospitals NHS Trust, Derby Hospital NHS Foundation trust, Bradford Teaching Hospitals NHS Foundation Trust, and the University Hospital of South Manchester NHS Foundation Trust). We thank the staff at the TISU trial office based in the Centre for Healthcare Randomised Trials (CHaRT) within the Health Services Research Unit, University of Aberdeen.
