# Supplementary material for: Shockwave Lithotripsy Versus Ureteroscopic Treatment as Therapeutic Interventions for Stones of the Ureter (TISU): A Multicentre Randomised Controlled Non-inferiority Trial[image]
Source: Eur Urol. 2021 Jul;80(1):46–54. doi: 10.1016/j.eururo.2021.02.044 (PMC8234516; doi:10.1016/j.eururo.2021.02.044)
Supplement: Supplementary file 1 [file mmc1.docx]

Supplementary Tables and Figures

Figures


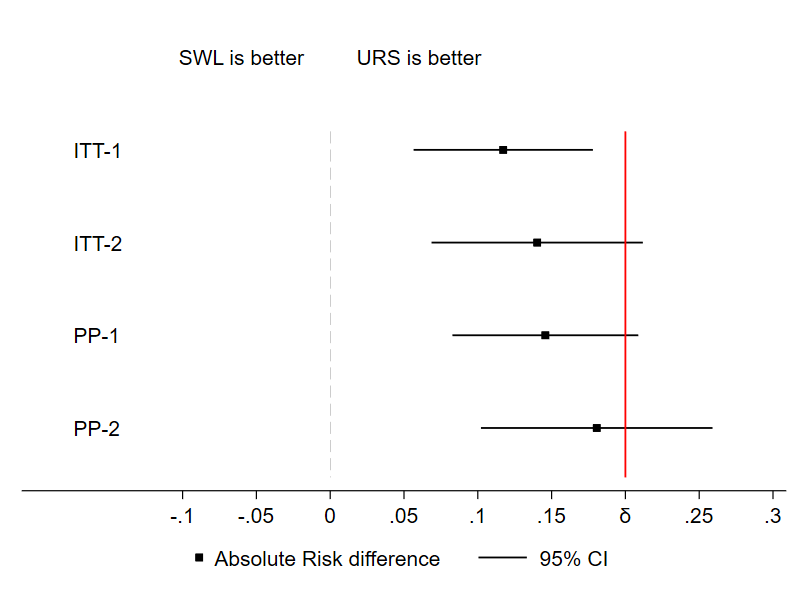


**Supplementary Figure 1** Absolute Risk Differences with 95% CI for the Primary Outcome, further treatment required under different analysis scenarios. ITT-1 Intention to treat including all participants; ITT-2 intention to treat but excluding those who passed their stone prior to any intervention. PP-1 per protocol including those that passed their stone before treatment; PP-2 per protocol analysis excluding those that passed their stone before treatment. The non-inferiority margin δ was set here at 0.2 (20%) as pre-specified in the protocol and sample size calculation.


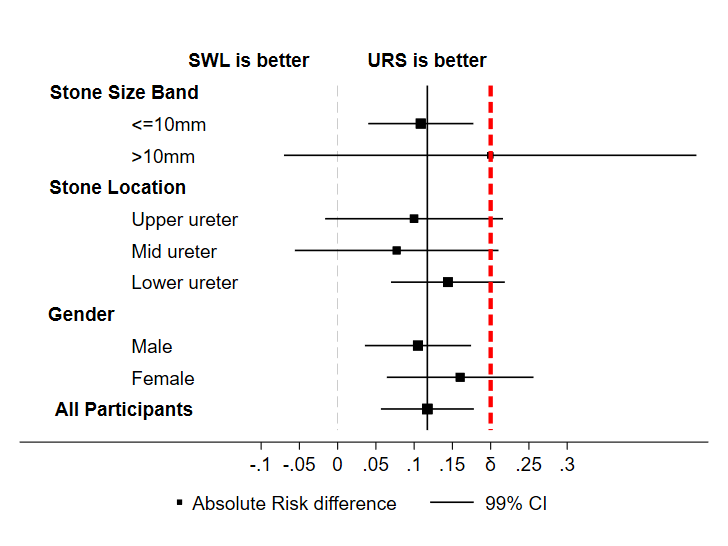


**Supplementary Figure 2** Absolute Risk Differences with 99% CI of a-priori sub-group analyses of the Primary Outcome, further treatment required. The non-inferiority margin δ was 0.2 (20%) as pre-specified in the protocol and sample size calculation.

# Supplementary Tables 1–3: Statistical Subgroup Analysis Models

Supplementary Table 1: Effect of SWL vs URS with Stone Size interaction

Stone size: ≤10mm vs >10mm

| Population | Main Treatment effect  Interaction effect | ARD^a,b^ | 95%CI | nonInf p-value | RR^a,b^ | 95%CI |
| --- | --- | --- | --- | --- | --- | --- |
| ITT-1 | SWL | 0.12 | (0.06, 0.18) | <0.001 | 2.11 | (1.27, 3.50) |
|  | Stone Size # SWL | 0.14 | (-0.17, 0.45) | 0.130 | 1.14 | (0.34, 3.83) |
| ITT-2 | SWL | 0.14 | (0.07, 0.21) | 1.000 | 2.208 | (1.34, 3.61) |
|  | Stone Size # SWL | 0.10 | (-0.21, 0.41) | 0.175 | 0.98 | (0.31, 3.13) |
| PP-1 | SWL | 0.15 | (0.08, 0.21) | <0.001 | 2.49 | (1.47, 4.21) |
|  | Stone Size # SWL | 0.18 | (-0.16, 0.52) | 0.168 | 1.10 | (0.32, 3.81) |
| PP-2 | SWL | 0.18 | (0.10, 0.26) | <0.001 | 2.66 | (1.58, 4.49) |
|  | Stone Size # SWL | 0.13 | (-0.22, 0.47) | 0.159 | 0.91 | (0.27, 3.02) |

ARD absolute risk difference; RR relative risk; ITT-1 Intention to treat including all participants; ITT-2 intention to treat but excluding those who passed their stone prior to any intervention. PP-1 per protocol including those that passed their stone before treatment; PP-2 per protocol analysis excluding those that passed their stone before treatment. nonInf p-value: non-inferiority p-value for the ARD results only

^a^ All treatment effect estimates adjusted for outcome at baseline, stone size, stone location, age, gender and centre as well as the (#) interaction being tested

^b^ Modified Poisson regression model with a log-link function and robust error variance

Supplementary Table 2: Effect of SWL vs URS with Stone Location interaction

Stone Loc1: middle vs upper ureter

Stone Loc2: lower vs upper ureter

| Population | Main Treatment effect  Interaction effect(s) | ARD^a,b^ | 95%CI | nonInf p-value | RR^a,b^ | 95%CI |
| --- | --- | --- | --- | --- | --- | --- |
| ITT-1 | SWL | 0.12 | (0.06, 0.18) | <0.001 | 1.63 | (0.92, 2.88) |
|  | Stone Loc1 # SWL | -0.01 | (-0.22, 0.20) | 0.030 | 1.48 | (0.29, 7.66) |
|  | Stone Loc2 # SWL | 0.05 | (-0.07, 0.17) | <0.001 | 2.36 | (0.92, 6.05) |
| ITT-2 | SWL | 0.14 | (0.07, 0.21) | <0.001 | 1.73 | (0.98, 3.05) |
|  | Stone Loc1# SWL | -0.02 | (-0.26, 0.23) | 0.051 | 1.47 | (0.28, 7.81) |
|  | Stone Loc2# SWL | 0.06 | (-0.08, 0.19) | 0.002 | 2.14 | (0.83, 5.52) |
| PP-1 | SWL | 0.15 | (0.08, 0.21) | <0.001 | 1.84 | (1.06, 3.20) |
|  | Stone Loc1# SWL | -0.01 | (-0.24, 0.21) | 0.041 | 1.81 | (0.30, 11.10) |
|  | Stone Loc2# SWL | 0.05 | (-0.08, 0.18) | 0.002 | 2.67 | (0.90, 7.92) |
| PP-2 | SWL | 0.18 | (0.10, 0.26) | <0.001 | 1.97 | (1.13, 3.44) |
|  | Stone Loc1# SWL | -0.01 | (-0.28, 0.25) | 0.068 | 1.84 | (0.30, 11.45) |
|  | Stone Loc2# SWL | 0.06 | (-0.10, 0.21) | 0.006 | 2.46 | (0.83, 7.33) |

ARD: absolute risk difference; RR: relative risk; ITT-1: Intention to treat including all participants; ITT-2 intention to treat but excluding those who passed their stone prior to any intervention. PP -1 per protocol including those that passed their stone before treatment; PP-2 per protocol analysis excluding those that passed their stone before treatment. nonInf p-value: non-inferiority p-value for the ARD results only

^a^ All treatment effect estimates adjusted for outcome at baseline, stone size, stone location, age, gender and centre as well as the (#) interaction being tested

^b^ Modified Poisson regression model with a log-link function and robust error variance

Supplementary Table 3: Effect of SWL vs URS with Gender interaction

Gender: Female vs Male

| Population | Main Treatment effect  Interaction effect | ARD^a,b^ | 95%CI | nonInf p-value | RR^a,b^ | 95%CI |
| --- | --- | --- | --- | --- | --- | --- |
| ITT-1 | SWL | 0.117 | (0.05, 0.18) | <0.001 | 1.85 | (1.20, 2.84) |
|  | Gender # SWL | 0.055 | (-0.05, 0.16) | <0.001 | 3.46 | (0.55, 21.67) |
| ITT-2 | SWL | 0.140 | (0.07, 0.21) | <0.001 | 1.94 | (1.27, 2.98) |
|  | Gender # SWL | 0.034 | (-0.10, 0.16) | 0.001 | 3.09 | (0.50, 19.14) |
| PP-1 | SWL | 0.146 | (0.08, 0.21) | <0.001 | 2.17 | (1.41, 3.34) |
|  | Gender # SWL | 0.052 | (-0.05, 0.16) | <0.001 | 3.22 | (0.53, 19.64) |
| PP-2 | SWL | 0.181 | (0.10, 0.26) | <0.001 | 2.32 | (1.51, 3.57) |
|  | Gender # SWL | 0.027 | (-0.10, 0.16) | 0.002 | 2.90 | (0.48, 17.33) |

ARD: absolute risk difference; RR: relative risk; ITT-1 Intention to treat including all participants; ITT-2 intention to treat but excluding those who passed their stone prior to any intervention. PP -1 per protocol including those that passed their stone before treatment; PP-2 per protocol analysis excluding those that passed their stone before treatment. nonInf p-value: non-inferiority p-value for the ARD results only

^a^ All treatment effect estimates adjusted for outcome at baseline, stone size, stone location, age, gender and centre as well as the (#) interaction being tested

^b^ Modified Poisson regression model with a log-link function and robust error variance
