# Supplementary material for: Shockwave Lithotripsy Versus Ureteroscopic Treatment as Therapeutic Interventions for Stones of the Ureter (TISU): A Multicentre Randomised Controlled Non-inferiority Trial[image]
Source: Eur Urol. 2021 Jul;80(1):46–54. doi: 10.1016/j.eururo.2021.02.044 (PMC8234516; doi:10.1016/j.eururo.2021.02.044)
Supplement: Supplementary file 2 [file mmc2.pdf]

TISU
Therapeutic Interventions for Stones of the Ureter

A multicentre randomised controlled trial of extracorporeal shockwave lithotripsy, as first treatment option, compared with direct progression to ureteroscopic treatment, for ureteric stones.

PROTOCOL

A UK Collaborative Study funded by the National Institute for Health Research Health Technology Assessment Programme (NIHR HTA)

Co-Sponsors

Name :	University of Aberdeen
Address:	Research and Innovation, Kings College, Regent Walk, Aberdeen AB24 3FX

Name :	Grampian Health Board
Address:	Research and Development Office, Foresterhill Annexe, Foresterhill, Aberdeen AB25 2ZD

Sponsor Number 3/087/12

Chief-Investigator

Name :	Professor Sam McClinton
Address:	NHS Grampian<br>Department of Urology, Ward 44, Aberdeen Royal Infirmary, Foresterhill, Aberdeen AB25 2ZB
Telephone:	01224 550517
Fax:	01224 550726
E-mail:	smcclinton@nhs.net

CI Signature:

Date:

Trial Office

Address:	TISU Trial Office<br>Centre for Healthcare Randomised Trials (CHaRT)<br>3rd Floor, Health Sciences Building<br>University of Aberdeen<br>Foresterhill<br>Aberdeen<br>AB25 2ZD
Telephone:	01224 438189
Fax:	01224 438165
E-mail:	tisu@abdn.ac.uk
Website:	http://www.charttrials.abdn.ac.uk/tisu

Other information:

International Standard Randomised Controlled Trial Number (ISRCTN): 92289221
REC Reference Number: 13/NS/2002

TABLE OF CONTENTS

PROTOCOL SUMMARY pp.5-7
Glossary of Abbreviations ....
Trial Personnel..

1. INTRODUCTION pp.8-9
1.1 Background to the study ....
1.2 Rationale for the study ....

2. STUDY OBJECTIVES pp.9-10

3. STUDY DESIGN...pp.10-15
3.1 Intervention to be evaluated ....
3.2 Study population...
3.2.1 Selection of Participants..
3.2.2 Planned Inclusion and Exclusion Criteria..
3.3 Recruitment and Study Procedures..
3.3.1 Identifying Participants...
3.3.2 Informed Consent..
3.3.3 Randomisation and Allocation..
3.3.4 Follow-Up Procedures..
3.3.5 Withdrawal Procedures..
3.3.6 Subsequent Arrangements..

4. SAFETY pp.15-16
4.1 Definitions ....
4.2 Recording and Reporting AEs and SAEs...

5. OUTCOME MEASURES p.16
5.1 Primary Outcome Measure...
5.2 Secondary Outcome Measure...

6. DATA COLLECTION AND PROCESSING pp.16-17
6.1 Measuring Outcomes......................
6.2 Schedule of Data Collection..
6.3 Data Processing...

7. SAMPLE SIZE, PROPOSED RECRUITMENT AND MILESTONES pp.17-18
7.1 Sample Size............
7.2 Recruitment Rates...
7.3 Milestones...
7.4 Feasibility Phase...
8. STATISTICAL ANALYSIS................................. pp.18-19

9. ECONOMIC EVALUATION.. ..pp.19-20
9.1 Collection of Data...
9.2 Participant Costs...
9.3 NHS Health Service Resource Use...
9.4 Cost Effectiveness...

10. ORGANISATION: TRIAL MANAGEMENT AND OVERSIGHT ARRANGEMENTS..........pp.20-21
10.1 Trial Office in Aberdeen...

10.2 Project Management Group (PMG)...
10.3 Trial Steering Committee (TSC)...
10.4 Data Monitoring Committee (DMC)..
10.5 Local Organisation in Sites...

11. RESEARCH GOVERNANCE, SPONSORSHIP AND DATA PROTECTION.................... pp.21-22
11.1 Research Governance..
11.2 Data Protection...
11.3 Sponsorship...

12. ETHICAL ISSUES AND ARRANGEMENTS......................... ....p.22

13. QUALITY ASSURANCE..p.22

14. FINANCE AND INSURANCE...p.22

15. DATA HANDLING, RECORD KEEPING AND ARCHIVING............................ ....p.22
1
6 SATELLITE STUDIES................. ....p.22
17. AUTHORSHIP AND PUBLICATION...p.23

REFERENCE LIST...p.24

APPENDICES.. ....pp.25-

PROTOCOL SUMMARY

**QUESTION ADDRESSED**	What is the clinical and cost-effectiveness of the use of extracorporeal shockwave lithotripsy (ESWL), compared to ureteroscopic treatment, as the first treatment option for symptomatic ureteric stones that require an active intervention?
**CONSIDERED FOR ENTRY**	Male and female patients aged ≥16 years with a ureteric stone confirmed by non-contrast computed tomography of the kidney, ureter and bladder (CTKUB).
**POPULATIONS**	Adults presenting with a ureteric stone within any segment of the ureter, confirmed by CTKUB, able to undergo either ESWL or ureteroscopy, where both the patient and clinician judge intervention to be necessary.
**STUDY ENTRY**	Consent to the RCT will be obtained from 750 eligible patients after written and oral information is provided by local hospital teams. Eligible and consenting participants will be randomised to one of the two intervention groups. Randomisation will be stratified by centre, stone size (<=10mm or >10mm, as measured by the maximum stone diameter on CTKUB) and location of the stone in either the upper, mid or lower ureter (as defined in the European Association of Urology-American Urological Association (EAU-AUA) Guidelines). Participants will be followed-up by postal questionnaires sent from the trial office (CHaRT, Aberdeen) at eight weeks and six months after randomisation and from their health record.
**INTERVENTIONS**	• Extracorporeal shockwave lithotripsy (ESWL)<br>• Ureteroscopic treatment
**OUTCOME ASSESSMENT**	The primary clinical outcome is clinical resolution of the stone episode (defined as no further intervention required to facilitate stone clearance) up to six months from randomisation.<br><br>The primary economic outcome is the incremental cost per quality adjusted life years (QALYs) gained at 6 months from randomisation. QALYs are based on the responses to the EQ-5D.
**CO-ORDINATION**	**Local**: by the local consultant Urologists, urology team and research nurses<br>**Central**: by Study Office in Aberdeen (Telephone 01224 438189)<br>**Overall**: by the Project Management Group, and overseen by the Steering Committee and the Data Monitoring Committee
**FUNDER**	The National Institute for Health Research (NIHR), Health Technology Assessment (HTA) Programme. Number 10/137/01
**Funding start date:**	1 March 2013
**Funding end date:**	28 February 2017

GLOSSARY OF ABBREVIATIONS	
AE	Adverse Event
BAUS	British Association of Urological Surgeons
CEAC	Cost-effectiveness Acceptability Curve
CHaRT	Centre for Healthcare Randomised Trials
CI	Chief Investigator / Confidence Interval
CRF	Case Report Form
CONSORT	Consolidated Standards of Reporting Trials
CTU	Clinical Trial Unit
CTKUB	Computed Tomography Scan of the Kidneys, Ureters and Bladder
DCE	Discrete Choice Experiment
DMC	Data Monitoring Committee
EAU-AUA	European Association of Urology-American Urological Association
eGFR	Estimated Glomerular Filtration Rate
ESWL	Extracorporeal Shockwave Lithotripsy
EQ-5D	EuroQol Group's 5 dimension health status questionnaire
GCP	Good Clinical Practice
GP	General Practitioner
HRQoL	Health Related Quality of Life
HSRU	Health Services Research Unit
HTA	Health Technology Assessment
ISD	Information Statistics Division
ISF	Investigator Site File
ISRCTN	International Standard Randomised Controlled Trial Number
IVR	Interactive Voice Response (randomisation)
NHS	National Health Service
NHSG	National Health Service Grampian
NIHR	National Institute Health Research
NRES	National Research Ethics Service
NRS	Numeric Rating Scale
PI	Principal Investigator
PIL	Patient Information Leaflet
PMG	Project Management Group
PQ	Participant Questionnaire
QALY	Quality-Adjusted Life Year
RCT	Randomised Controlled Trial
R&D	Research and Development
REC	Research Ethics Committee
SAE	Serious Adverse Event
SAP	Statistical Analysis Plan
SD	Standard Deviation
TMF	Trial Master File
TSC	Trial Steering Committee
VDS	Verbal Descriptor Scale
UK	United Kingdom
UKCRC	United Kingdom Clinical Research Collaboration
UoA	University of Aberdeen

TRIAL PERSONNEL

Chief Investigator

1	Sam McClinton<br>(Consultant Urologist)

Grant Holders

1	James N'Dow (Consultant Urologist)	8	John Norrie (CHaRT Director)
2	Graeme MacLennan (Lead Statistician)	9	Sarah MacLennan (Health Psychologist)
3	Mary Kilonzo (Health Economist)	10	Ruth Thomas (Research Manager)
4	Frank Keeley (Consultant Urologist)	11	Kath Starr (Trial Manager)
5	Ken Anson (Consultant Urologist)	12	Neil Burgess (Consultant Urologist)
6	Charles Clark (Patient Group Advisor)	13	Thomas Lam (Consultant Urologist)
7	Robert Pickard (Consultant Urologist)	14	Lufti Kurban (Consultant Radiologist)

Project Management Group (PMG)

This group is comprised of the grant holders along with representatives from the TISU central study team:

1	Trial Manager	5	Senior trials manager
2	Data co-ordinator	6	Senior IT manager
3	Health economist	7	
4	Statistician	8	

Trial Steering Committee (TSC) Members

This committee is comprised of four independent members along with Chief Investigator (Sam McClinton), the other TISU grant-holders and key members of the central office (eg trial manager) may attend TSC meetings. The funders will be notified in advance of meeting and a representative invited to attend. Other relevant experts may be invited to attend as appropriate.

Independent Members

1	Roger Kockelbergh (Chair)	3	Sarah Meredith
2	John McGrath	4	Stanley Coutts

Data Monitoring Committee (DMC) Members

1	Elaine McColl (Chair)	3	Richard Emsley
2	Simon Harrison	4	

Trial Office Team

This team comprises of the CI, Aberdeen-based grant holders and central office team members.

1. INTRODUCTION

1.1. Background

Urinary stone disease is very common with an estimated prevalence among the general population of 2–3% (1.8 million people in the UK) with males forming stones three times as often as females.[1] Urinary stones often recur and the lifetime recurrence rate is approximately 50%.[2] The interval between recurrences is variable, with approximately 10% within one year, 35% within five years, and 50% within 10 years. [3] The increased incidence of urinary stones in the industrialised world is associated with improved standards of living (mainly due to the high dietary intake of proteins and minerals) and there is also an association with ethnicity and region of residence. [4] Urinary tract stones, and ureteric stones, in particular, are associated with severe pain as they pass through the urinary tract and can have a significant impact on patients' quality of life due to the detrimental effect on their ability to work and the need for hospitalisation.

Urinary stones are a major burden on the NHS resulting in over 84,323 finished consultant episodes and over 97,558 bed-days in England in 2011 - 2012.[5] When urinary stones move from the kidney into the ureter (tube connecting the kidney to the bladder), they cause severe debilitating pain (ureteric colic) that causes a large transient impairment of quality of life and leads to substantial calls on health service resources. Ureteric colic is the most common cause of emergency admission to Urology departments in the UK [5] and since it predominantly affects younger people (16-55 years) is a common cause of time off work. The aim of treatment for ureteric stones is the immediate relief of symptoms, decompression of the urinary tract and the achievement of clinically complete stone clearance.

Most ureteric stones can be expected to pass spontaneously with supportive care (painkillers and fluids) possibly aided by drugs such as alpha blockers or calcium channel blockers (conservative management). The clinical effectiveness and cost effectiveness of such drug therapy in facilitating stone clearance remains unclear and is the subject of an ongoing HTA commissioned randomised controlled trial led by this research group (due to report in 2014). However, between a fifth and a third of cases require an active intervention (stone removal) because of failure to pass the stone, continuing pain, infection or obstruction to urine drainage. The two standard active intervention options are extracorporeal shockwave lithotripsy (ESWL) and ureteroscopic stone retrieval. Whilst both ESWL and ureteroscopy appear to be effective in terms of stone clearance they differ in terms of invasiveness, anaesthetic requirement, treatment setting, the number of procedures required to clear the stone, complications, patient reported outcomes (such as severity and duration of pain after intervention, time off work and bothersome urinary symptoms), and cost. There is uncertainty around which is the most clinically effective in terms of stone clearance and the true cost to the NHS and to society (in terms of impact on patient reported health and economic burden).

A joint clinical guideline on the management of ureteric stones by the European Association of Urology and the American Urological Association[6] estimates that 68% of stones ≤5 mm and 47% of stones 5-10mm in size can be expected to pass spontaneously and concluded that the majority of these stones pass within four to six weeks of presentation. Stones in the distal ureter pass more readily than stones located more proximally. Consequently, patients with favourable features and with smaller sized stones in the lower ureter are initially treated conservatively. Immediate active intervention occurs in those patients with larger stones and unfavourable features who are deemed clinically to be unsuitable for conservative treatment. Those who fail standard conservative care or who subsequently develop complications also undergo later active treatment. This can be extra-corporeal shock wave lithotripsy (ESWL), preliminary ureteric stenting with later stone removal, ureteroscopy with stone retrieval or destruction (in situ lithotripsy), or percutaneous nephrostomy insertion and later stone removal. ESWL and ureteroscopic treatment require expensive equipment and urological expertise. Both have been shown to be options that are safe and effective in a number of studies. In clinical practice urologists tend to favour ureteroscopy over ESWL particularly for mid and lower ureteric stones due to perceived higher rate of clinical stone clearance.[7]

1.2. Rationale for the study

A Cochrane systematic review (2007)[8] comparing the effectiveness of ESWL with ureteroscopic management of ureteric stones identified five RCTs involving a total of 732 participants. The results of this review suggest stone-free rates were lower in the ESWL group (RR 0.83 95% CI 0.70 to 0.98) and reflecting this retreatment rates for ESWL were higher (RR 2.78 95% CI 0.53 to 14.71) but these findings are associated with much uncertainty. Complications were less frequent after ESWL (RR 0.44 95% CI 0.21 to 0.92) and this option was associated with a shorter hospital stay (mean difference -2.10 days 95% CI -2.55 to -1.64 days). The review concluded that ureteroscopic treatment of stones was associated with a higher stone free rate but a higher morbidity (complication rate) and a longer hospital stay. However, the overall quality of the studies was poor and inclusion criteria were strict in each study, limiting both the generalisability and applicability of the review findings. There was limited evidence on which to judge the comparative effectiveness in clinically important prognostic subgroups for example location of stones in the ureter. None of the studies reported on health related quality of life and only one reported a cost effectiveness outcome. The review authors recommended that a large scale multicentre RCT was needed to adequately address the effectiveness and cost effectiveness of ureteroscopy versus ESWL. A 2011 update of the Cochrane review [9] has included two further studies but these have not altered the previous conclusions.

The European Association of Urology and the American Urological Association (EAU-AUA) Clinical Guideline Panel on the management of ureteric stones also conducted a review and meta-analysis reporting clinical outcomes and complications following treatment of ureteric stones with ESWL or ureteroscopy, including data from non-randomised comparisons and case series.[6] Pre-defined outcome measures were stone-free rate and number of additional procedures required. The results were stratified according to stone location in the ureter (proximal, mid, distal) and stone diameter (<=10mm, >10mm). All forms of ESWL were analysed as a single treatment modality in the meta-analysis. The Panel concluded that the main advantage of ureteroscopy is a higher stone-free rate with a single procedure, but with a higher complication rate. However, the evidence was insufficient for the Panel to recommend between ESWL and ureteroscopy and concluded that for patients requiring active stone removal, either treatment modality is acceptable as first-line options. For the individual patient, the choice is often determined by a number of factors including the availability of resources, preference of the treating urologist, and preference of the patient. The review highlighted design and reporting deficiencies from available studies, including poor definition of stone size, inconsistent reporting of outcomes and lack of randomisation. One of the main recommendations of the Panel was the need to conduct randomised controlled trials (RCTs) comparing the clinical and cost effectiveness of ESWL and ureteroscopy.

In response to the research questions raised by these evidence summaries, the TISU trial will provide the high quality evidence, from a large pragmatic RCT, on the relative effectiveness, and cost effectiveness of ESWL and ureteroscopy, that will inform patients, clinicians and policy makers on the optimal choice of intervention for ureteric stones.

2. STUDY OBJECTIVES

The research question that we will address is whether in adults with ureteric stones judged to require active intervention, extracorporeal shockwave lithotripsy (ESWL) is more effective and cost-effective compared to ureteroscopic treatment as the initial management option.

The hypothesis being tested is that outcome in patients receiving ESWL as their first treatment option is not inferior to outcome in patients receiving direct ureteroscopic retrieval.

The clinical and cost-effectiveness of ESWL, as the first treatment option compared with direct progression to ureteroscopic treatment will be determined with respect to the primary outcomes of:

i. Clinical stone clearance, defined as no further intervention required to facilitate stone passage
ii. incremental cost per quality adjusted life years (QALYs) and
iii. disease or treatment-related harms up to 6 months after randomisation.

These outcomes will be measured by:

- The clinical effectiveness of ESWL compared with ureteroscopic treatment in terms of proportion of patients who do not require any further intervention at 6 months
- The cost-effectiveness of a treatment strategy that starts with ESWL compared with one that starts with ureteroscopic treatment at 6 months

A non-inferiority design has been chosen as ESWL offers a less invasive approach, avoiding general anaesthetic, which might avoid the extra cost and more invasive nature of ureteroscopic treatment. If ESWL fails then patients can proceed to ureteroscopy. Our patient group and urologists, who are members of the BAUS Section of Endourology, when asked stated they would prefer the less invasive treatment option and would accept a higher failure rate (up to 20%) and the higher risk of reintervention.

The definitive clinical measure of the effectiveness of ESWL and ureteroscopic treatment for the management of urinary stones is whether the patient's stone is cleared and any symptoms resolved. Current evidence and estimates of the effectiveness of the interventions for management of urinary stones are based upon "stone clearance" as an outcome. However, to assess stone clearance with certainty would require that all patients have repeated computed tomography scans of the kidneys, ureters, and bladder (CTKUB) until the stone is no longer imaged. This is not standard practice because it results in radiation exposure and is unlikely to change patient management. For our primary outcome we have defined stone clearance as no further intervention required to facilitate stone clearance up to 6 months after randomisation. The decision on the necessity of a further intervention (primary outcome) is that of the treating clinician unblinded to the treatment received and so is potentially open to bias based upon the treating clinician's knowledge and perceptions of the efficacy of the intervention. To minimise this possibility the decision that further intervention is required will be based on EAU-AUA guidelines.

3. STUDY DESIGN

A pragmatic multicentre non-inferiority randomised controlled trial of ESWL as first treatment option compared with direct progression to ureteroscopic treatment for ureteric stones (incorporating an internal feasibility phase). A summary of the trial design is shown in Figure 1.

3.1. Intervention to be evaluated

- Extracorporeal shockwave lithotripsy (ESWL)
- Ureteroscopy

Extracorporeal shockwave lithotripsy (ESWL) involves generation of a shock-wave, external to the body, focused on the stone, causing it to fragment with the fragments subsequently passing spontaneously. A variety of systems (differing means of generating shock-waves, different focusing mechanisms) are available. It is routinely performed in an outpatient setting with pain relief or sedation as required. Recruitment will occur only in established centres with fixed-site lithotripters. This will allow some standardisation of protocols on times to treatment and ESWL delivery. Up to two sessions of ESWL will be considered as one intervention as per standard practice.

Ureteroscopy is the use of small semi-rigid or flexible ureteroscopes, in conjunction with intracorporeal lithotripsy devices, such as the holmium laser, to directly visualise and fragment ureteric stones. Smaller stones, in the lower ureter, can often be removed intact using basketing

devices. It is currently most often performed as a day-case procedure (but may require hospital admission depending on complexity) and usually requires general anaesthesia.

All post allocated treatment, auxiliary or additional procedures and interventions will be used as and when clinically indicated based on EAU/AUA and standard care guidelines. The reasons why further intervention may be required are similar across both arms. The decision about which further treatments are given will be influenced by the treatments that participants have already received and the need to treat any complications that may arise

Further interventions may include:

1. Extracorporeal shockwave lithotripsy (ESWL);
2. Urgent/elective ureteroscopy with stone fragmentation
3. Endoscopic insertion (or removal) of a stent in the ureter;
4. Percutaneous insertion of nephrostomy
5. Antegrade insertion of a ureteric stent through a nephrostomy

Figure 1 Flow Diagram: Randomised controlled trial comparing extracorporeal shockwave lithotripsy (ESWL) with ureteroscopic retrieval as first treatment options for urinary stones.

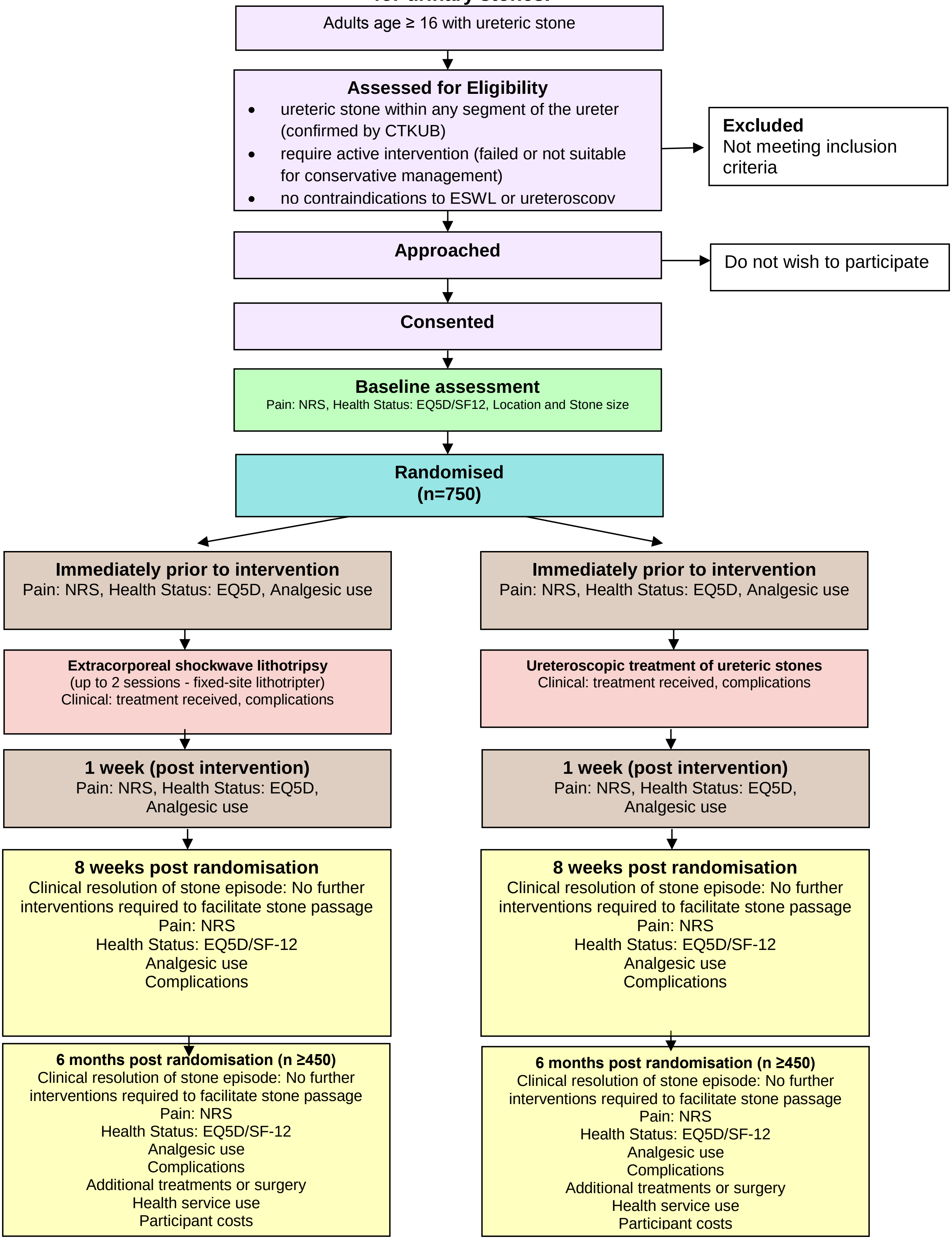

3.2 Study population

Adults (≥ 16 years old), presenting with a unilateral ureteric stone within any segment of the ureter, confirmed by computed tomography scan of the kidneys, ureters, and bladder (CTKUB), who are judged clinically to require active intervention and are able to undergo either treatment, capable of giving written informed consent, which includes adherence with the requirements of the trial

3.2.1 Selection of participants

As standard practice, clinicians will assess patients presenting with a suspected ureteric stone A log will be taken of all patients assessed in order to document the reasons for non-inclusion in the study (e.g. reason they were ineligible, or declined to participate) to inform the CONSORT diagram.

Brief details of potentially eligible patients will be recorded in the screening logs at each site (these will be an aid to monitoring potential participant inclusion).

3.2.2 Planned inclusion and exclusion criteria

Inclusion criteria

- Presence of stone confirmed by CTKUB
- Patients with a ureteric stone requiring removal
- Adults ≥16 years of age
- Single ureteric stone requiring treatment
- Suitable for either ESWL or ureteroscopic treatment
- Capable of giving written informed consent, which includes adherence with the requirements of the trial.

Exclusion criteria

- Pregnancy
- Stones not confirmed by CTKUB
- Bilateral ureteric stone(s)
- Patients with abnormal urinary tract anatomy (such as a horseshoe kidney or ileal conduit)
- Patients unable to understand or complete trial documentation

3.3 Recruitment and Study Procedures

3.3.1 Identifying participants

Local procedures at the participating hospitals are different and the timing and mode of approach to patients and the consent process will vary to accommodate both the variability at the sites and the needs of the patients. Following adequate pain relief and confirmation of ureteric calculi by CTKUB, eligible patients (according to the inclusion and exclusion criteria given in sections 3.2.2) will be provided with a patient information leaflet (PIL).

Each patient will have the opportunity to discuss the study with the local clinical team. Patients may make a decision to participate, during a consultation with the local clinical team during a visit to hospital (e.g. when they attend a clinic appointment or whilst a patient in hospital for their initial stone episode) or alternatively at home. If the patient agrees to be contacted at home he/she may receive a telephone call from the local Research Nurse to discuss any queries. Patients who decide to participate following telephone counselling can either send their completed documents (consent form and baseline questionnaire) through the post to the local team at their treating hospital or bring it with them if they are returning to hospital for another consultation or treatment. Participants will be

randomised to one of the two treatment groups following consent, completion and receipt of baseline questionnaire.

3.3.2 Informed consent

The PIL explains that the trial is investigating the use of either ESWL or ureteroscopic treatment as the first treatment option for symptomatic ureteric stones that require an active intervention. Signed informed consent forms will be obtained from the participants in all centres. Participants who cannot give informed consent (e.g. due to incapacity) will be not be eligible for participation. The participant's permission will be sought to inform their general practitioner that they are taking part in this trial.

3.3.3 Randomisation and allocation

Eligible and consenting participants will be randomised to one of the two intervention groups using the telephone Interactive Voice Response (IVR) randomisation application or via the web based application - both hosted by the fully registered with the UK Clinical Research Collaboration (UKCRC), Clinical Trials Unit (CTU) at the Centre for Healthcare Randomised Trials (CHaRT), Health Services Research Unit (HSRU) in Aberdeen.

Randomisation will be minimised by centre, stone size (<=10mm or > 10mm, as measured by the maximum stone diameter on CT KUB) and location of the stone in either the upper, mid or lower ureter (as defined in the EAU/AUA Guidelines).

3.3.4 Follow-up procedures

Eligible patients that have given signed informed consent to participate in the study will be asked to complete the EQ-5D, SF-12, pain score (NRS), use of analgesics at baseline and will then be randomised to either ESWL or ureteroscopic retrieval. Participants will be asked to complete the pain score, EQ5D, and use of analgesics questions (self completed) at their allocated intervention visit to secondary care immediately prior to receiving their interventions. At one week post intervention they will be asked to complete the pain score and use of analgesics questions by self completed questionnaire. Participants allocated to ESWL (up to two sessions of ESWL) will receive these questionnaires for their first session of ESWL. At eight weeks and then six months post randomisation participants will be asked to complete a questionnaire to measure the EQ-5D, SF-12, pain score (NRS), use of analgesics, complications, additional interventions received, and acceptability of the received procedure. In addition, at six months post randomisation, participants will be asked to complete questions relating to their primary and secondary care. Questionnaires and up to two reminders will be sent to participants by post, email or phone, taking into account any preferences they may have for mode of communication.. The case report forms (CRFs) at eight weeks and six months post randomisation will be completed and entered at site by the centre coordinators at the recruiting centres. They will collect additional interventions received and reasons for those, reasons why they might not have received their allocated intervention, complications and date of stone passage.

3.3.5 Withdrawal procedures

Participants will remain on the trial unless they chose to withdraw consent or if they are unable to continue for a clinical reason. If a participant withdraws consent, participant questionnaires will not be collected, however permission will be sought for the research team to continue to collect outcome data from their health care records (via the CRFs). All other changes in of status with the exception of formal withdrawal of consent will mean the participant is still followed up for all study outcomes wherever possible.

3.3.6 Subsequent arrangements

Informing key people
Following formal trial entry:

The Study Office will:

i) Inform the participant's General Practitioner (by letter enclosing information about TISU and Study Office contact details).

The local Research Nurse will:

i) File the Hospital Copy of the Consent form in the hospital notes along with information about TISU.
ii) Use the TISU internet database to enter data regarding the participant, including data required to complete randomisation
iii) Return all study documentation to the Study Office in Aberdeen after database entry of essential data.

Notification by GPs
GPs are asked to contact the Study Office if one of the participants moves, becomes too ill to continue or dies, or any other notifiable event or possible serious adverse event occurs. Alternatively, staff at the Study Office may contact the GP.

4 SAFETY

The TISU trial involves procedures for treating ureteric stone which are well established in clinical practice. Adverse effects may occur during or after any type of surgery.

4.1 Definitions

An adverse event (AE) is defined as any untoward medical occurrence in a participant, not necessarily having a causal relationship.

Adverse events are not:

- continuous and persistent disease or symptom, present before the trial, which fails to progress;
- signs or symptoms of the disease being studied (in this case ureteric stones); or
- treatment failure.

An adverse event is defined as “serious” (SAE) if it

- Results in death
- Is life threatening
- Requires or prolongs inpatient hospitalisation
- Results in persistent/significant disability/incapacity
- Is otherwise considered medically significant by the investigator.

Expected adverse events:
In this study the following events are potentially expected:

ESWL: bleeding on passing urine, pain, urinary tract infection, bruising of abdomen/loin skin, stone fragments stuck between kidney and bladder, infection, kidney damage and persistence of stones.

Ureteroscopy: burning or bleeding on passing urine, temporary insertion of bladder catheter, insertion of stent and further procedure to remove it, pain, inability to retrieve stone, movement of stone into kidney, kidney damage or infection, failure to pass the telescope, recurrence of stones, damage to ureter, scarring of ureter.

4.2 Recording and reporting AEs and SAEs

Assessing and recording AEs and SAEs

Non-serious events will not be collected or reported. Planned hospital visits for conditions other than those associated with the ureteric stone will not be collected or reported. Hospital visits (planned or unplanned) associated with further interventions to facilitate ureteric stone clearance will be recorded as an outcome measure, but will not be reported as serious adverse events.

Within TISU, 'relatedness' is defined as an event that occurs as a result of a procedure required by the protocol, whether or not this procedure is the specific intervention under investigation and whether or not it would have been administered outside the study as normal care.

Any SAEs related to the participants' ureteric stone treatment that are not further interventions to facilitate stone clearance (eg if a participant is admitted to hospital for treatment of infection) will be recorded on the serious adverse event form. In addition all deaths for any cause (related or otherwise) will be recorded on the serious adverse event form.

Reporting responsibilities of the CI
When an SAE form is uploaded onto the trial website, the Trial Manager will be automatically notified. If, in the opinion of the local PI and the CI, the event is confirmed as being *serious* and *related* and *unexpected*, the CI or Trial Manager will notify the sponsor within 24 hours of receiving the SAE notification. The sponsor will provide an assessment of the SAE. The CI (or Trial Manager) will report any related and unexpected SAEs to the main REC and the DMC within 15 days of the CI becoming aware of it. All related SAEs will be summarised and reported to the Ethics Committee, the Funder and the Trial Steering Committee in their regular progress reports.

5 OUTCOME MEASURES

5.1 Primary outcome measure
The study has a primary clinical and a primary economic outcome reflecting the multidimensional nature of the possible effects the intervention may have.

Clinical Clearance of ureteric stones operationally defined as "no further intervention required to facilitate stone clearance" up to 6 months from randomisation.

Economic Incremental cost per quality adjusted life years (QALYs) gained at 6 months from randomisation. QALYs are based on the responses to the EQ-5D.

5.2 Secondary outcome measures
Patient-reported -measured at pre and 1 week post intervention, 8 weeks and 6 months post randomisation

Severity of pain as measured by the Numeric Rating scale (NRS)[10], generic health profile as measured by the SF-12, (8 weeks and 6 months only), health status as measured by the EQ5D,use of analgesia, acceptability of received procedure (8 weeks and 6 months only)

Clinical Further interventions received, complications up to 6 months post randomisation

Economic NHS primary and secondary care use and costs up to 6 months, patient costs; incremental cost per surgical interventions averted.

6. DATA COLLECTION AND PROCESSING

6.1. Measuring outcomes

Measurement and timing of outcome assessment (see Table 1: Source and timing of measures)

Outcome data will be collected throughout the trial from consent until six months following randomisation. See Table 1 for detail.

6.2. Schedule of data collection

Table 1: Source and timing of measures

Outcome measures	Source	Timing				
			Intervention		Post randomisation	
		Recruitment	Pre	1 week post	8 weeks	6 months
Additional interventions received	CRF & PQ				✓	✓
Pain (NRS)	PQ	✓	✓	✓	✓	
Health status EQ5D	PQ	✓	✓	✓	✓	✓
Health profile SF-12	PQ	✓			✓	✓
Use of analgesics	PQ	✓	✓	✓	✓	
Complications	CRF				✓	✓
NHS primary and secondary health care use	PQ & CRF					✓
Participant costs	PQ					✓

6.3 Data Processing

Research Nurses will enter locally-collected data in the centres. Staff in the Study Office will work closely with local Research Nurses to ensure that the data are as complete and accurate as possible. Follow up questionnaires to participants will be sent from and returned to the Study Office in Aberdeen. Extensive range and consistency checks will further enhance the quality of the data.

7. SAMPLE SIZE, PROPOSED RECRUITMENT RATE AND MILESTONES

7.1 Sample size

The original sample size calculations reflect that the TISU trial is a non-inferiority design. Published literature ([6,8,9]) suggests the proportion stone free without further intervention up to six months in the ureteroscopy arm will be about 0.75 (P1) and in the ESWL arm about 0.65 (P2). The margin of inferiority deemed acceptable is 0.20 so that P2-P1> -0.20. The sample size was estimated using simulations, designed for this purpose, run in Stata. The power of a non-inferiority trial can be considered as the probability that the lower bound of the estimated confidence interval (CI) around the difference between trial proportions

excludes the margin of non-inferiority. Simulating 1000s of trials of fixed sizes with the parameters P1 and P2 as above indicates that a trial of 450 per arm is required for the lower bound of the estimated 95% CI to exclude -20% with 90% power. Adjustment for potential 10% drop-out inflates the trial to 1000 in total. A trial of this size would have above 90% power to test superiority on secondary outcomes of an effect size of 1/4 of a standard deviation.

Following poor recruitment and interim data analysis of 267 participants the sample size was amended from 1000 downwards to 750. The amendment has been ratified by the trial oversight committees and the sponsor and the funder. Recruitment projections showed us that the original sample size of 1000 was unachievable in a realistic timeframe despite measures implemented to improve recruitment. We agreed with the funder an extension of 18 months to reach a revised sample size of 750. Our original sample size of 1000 included a 15% uplift from 850 to perform the primary analysis under a suitably defined Per Protocol analysis. A Per Protocol analysis, in the special context of a non-inferiority design is often seen as the more conservative approach than the conventional intention to treat analysis.

This Per Protocol analysis would have excluded any participants that crossed over from their randomly allocated treatment to the other, and would have excluded any participant whose stone cleared before their randomly allocated treatment was initiated. However, the view of the HTA Board, which has since been confirmed by the TISU iDMC, is that the intention to treat (ITT) approach should take primacy over this Per Protocol approach, to better reflect that the purpose of this pragmatic effectiveness trial is to compare the policy of initiating one or other of these treatment options, and not to focus on the actual comparative performance of the treatment options themselves. The Per Protocol approach will still be used as a supporting analysis. This is also a rare context in which there is no missing outcome data, since we are sure that we will have identified any further interventions to facilitate stone clearance in the 6 months post operatively - hence we can say with certainty for each individual randomised whether the original operation cleared the stone.

Based on the 267 participants with mature outcome data as of the 16 February 2016, the iDMC observed that all the assumptions behind the power calculation remain plausible. Given that we are now committed to the ITT approach rather than the Per Protocol approach (and under, the original design of assuming the proportion stone free in ESWL arm would be 65%, and 75% in the ureteroscopy arm) the achievable sample size of 750 will give 85% power. If we recruit 750, under the assumption of 15% crossover and stone clearance, prior to initiation of the randomly allocated intervention, then the per protocol analysis of 638 subjects will still retain ~80% power under the same assumptions as above.

7.2 Recruitment rates (See Figure 2: Projected participant recruitment and centre start up graph)

7.2.1 Original Recruitment rates

Across approximately 17 NHS centres, each serving populations of around 0.75 million, we aim to recruit an average of 3-4 per month per centre to achieve the target of 1000 participants over a 29 month recruitment period. The graph in Figure 2 illustrates the original projected participant recruitment and centre start up.

7.2.2 Updated Recruitment rates

Recruitment has been ongoing for 36 months and will continue for another 12 months. We aim to recruit a new target of 1 participant per month per centre across approximately 22 NHS centres to achieve the revised target of 750 participants. The graph in Figure 3 illustrates the revised projected participant recruitment.

Figure 2: Projected participant recruitment and centre start up graph

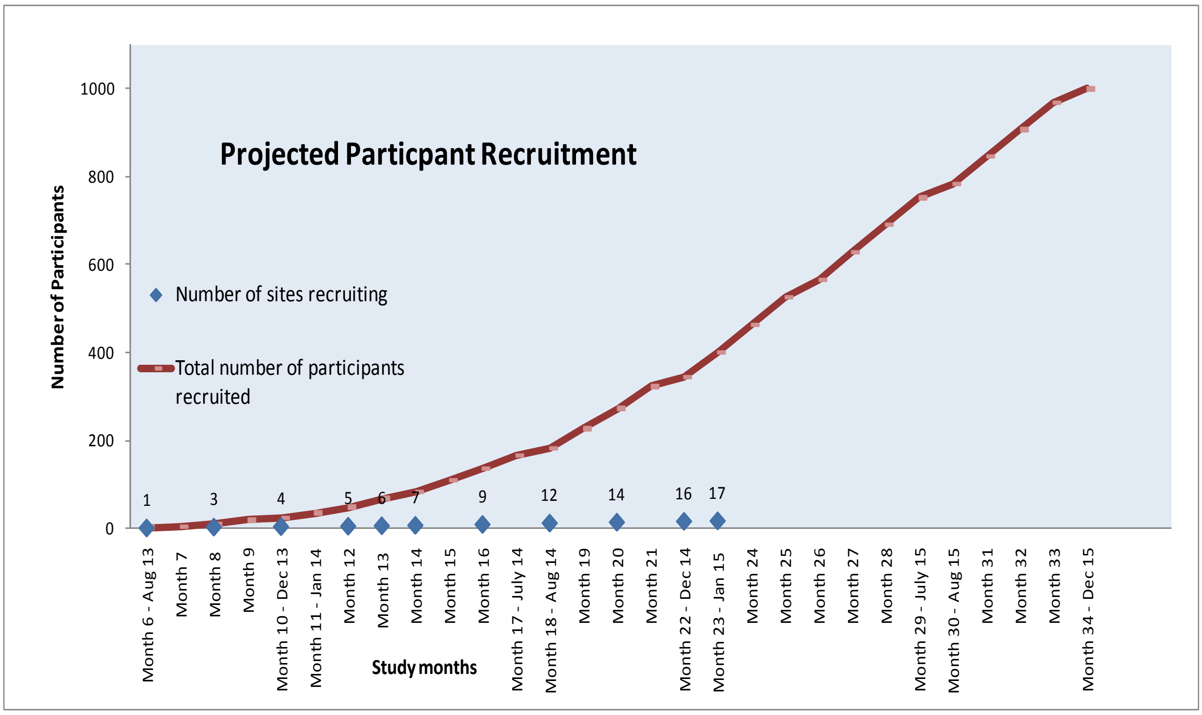

Figure 3: Revised projected participant recruitment

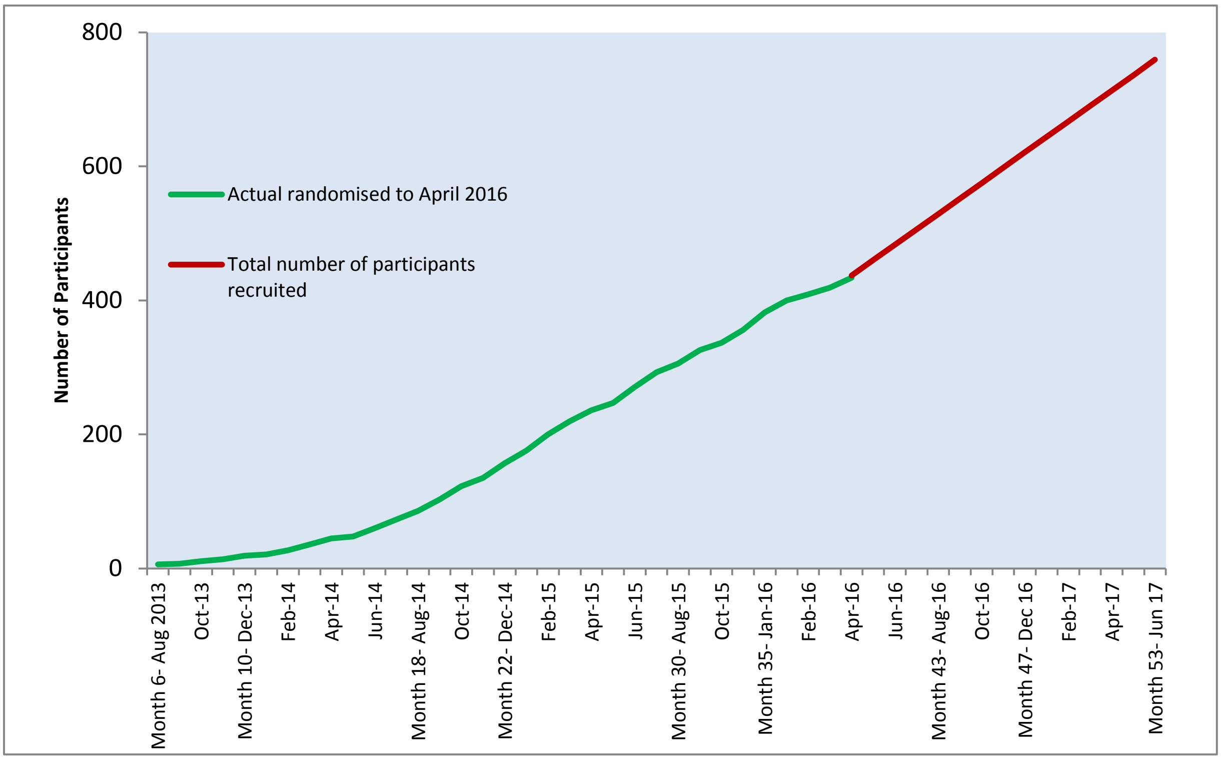

7.3 Milestones

The project timetable and milestones can be found in Appendix 1.

7.4 Feasibility phase

The internal feasibility phase will assess the key trial delivery parameters. The feasibility phase will

- Determine whether the proposed rate of recruitment is feasible;

- Monitor whether the allocated interventions are delivered within 8 weeks of randomisation;
- Finalise the optimum mode and timing of acute pain measurements.

The feasibility phase will be run as per any large multi-centre randomised trial. The feasibility phase will conclude with at least 23 centre months aggregated across at least three sites (with the expectation of at least 60 participants). This will present one of three situations:

1. Recruitment is within 75% of the target, in which case there is sufficient reassurance to continue unchanged to the main study
2. Recruitment is between 50% and 75% of the target rate, in which case the feasibility phase will continue with appropriate modifications (such as recruiting more centres, or allowing for more recruitment time at a centre)
3. Recruitment is less than 50% of the anticipated rate, in which case the study may not be feasible.

8. STATISTICAL ANALYSIS

Treatment groups will be described at baseline and follow-up using means (with standard deviations), medians (with inter-quartile ranges) and numbers (with percentages) where relevant. Primary and secondary outcomes will be compared using generalised linear models, with adjustment for participant baseline and design covariates, (stone <= 10 mm and stone >10mm; location in ureter: upper, mid, or lower; age; and gender).

Statistical analysis will be per protocol and intention-to-treat (as is recommended for non-inferiority trials) with results displayed as estimates and 95% confidence intervals (CIs) derived from appropriate generalised linear models. CIs around observed differences will then be compared to the pre-specified non-inferiority margin. Subgroup analyses (appropriately analysed by testing treatment by subgroup interaction) will explore the possible effect modification by type and location of stone and gender; all using stricter levels of statistical significance ($p<0.01$, 99% confidence intervals).

All analyses will follow a carefully documented Statistical Analysis Plan (SAP). The SAP will be available to both the Trial Steering Committee (TSC) and the independent Data Monitoring Committee (DMC) members should they wish to review and comment on the document. A single main analysis will be performed at the end of the trial when all follow up has been completed.

9. ECONOMIC EVALUATION

Economic evaluation will be an integral part of the study. Resource use and costs will be estimated for each participant. The evaluation will consider the costs of the care pathways that patients receive. Resource data collected will include the costs of the interventions, ESWL and ureteroscopy, and simultaneous and consequent use of primary and secondary NHS services (including additional interventions received) by participants. Personal costs such as purchase of medications, particularly analgesics, time and travel will also be estimated. The perspective of the study will be societal as it will include both the NHS costs as well as that of the participants.

9.1 Collection of data

Primary and secondary care resource use will be collected via the case report forms (CRF) and participant questionnaires. Unit costs will be based on routine sources (e.g. Reference Costs or study specific estimates. QALYs will be based on the responses to the EQ-5D.

9.2 Participant costs

Participant costs will include self-purchased healthcare: such as prescription costs and over the counter medications particularly analgesics.

9.3 NHS health service resource use

Use of secondary care services following the treatment period will be collected using participant questionnaires and CRF. Information on outpatient visits, readmissions relating to the use and consequences of the interventions being compared will be recorded. Use of primary care services such as prescription medications, contacts with primary care practitioners e.g. GPs and practice nurses will be collected via the ‘health care utilisation questions’ administered at 6 months follow-up.

9.4 Cost effectiveness

The cost effectiveness will be measured in terms of costs of the treatment care pathways and quality adjusted life years (QALYs) at six months. QALYs will be estimated by transforming the EQ5D scores (collected at baseline, eight weeks and six months post-randomisation) into utility values using standard algorithms.11 The results will be presented as point estimates of mean costs, QALYs, and incremental cost per QALY of each treatment care pathway. Measures of variance for these outcomes are likely to involve bootstrapping estimates of costs and incremental QALYs. Incremental cost-effectiveness data will be presented in terms of cost-effectiveness acceptability curves (CEACs). Forms of uncertainty, e.g. concerning the unit cost of resources from the different centres, will be addressed using standard deterministic sensitivity analysis. Sensitivity analysis will also be used to explore the impact of statistical imprecision and other forms of uncertainty. Where feasible the results of the sensitivity analyses will also be presented as CEACs.

10. ORGANISATION: TRIAL MANAGEMENT AND OVERSIGHT ARRANGEMENTS

10.1 Trial office in Aberdeen

The Study Office is in CHaRT, Health Services Research Unit (HSRU) in Aberdeen and provides day to day support for the clinical centres. It is responsible for all data collection (such as mailing questionnaires), follow-up, data processing and analysis. It is also responsible for providing and maintaining the randomisation service, and communicating with the sites about TISU specific issues. We will produce newsletters for collaborators to inform everyone of progress and maintain enthusiasm.

The TISU Study Office Team (Aberdeen-based grant holders and study office members) will meet at least monthly during the course of the study to ensure smooth running and trouble-shooting.

10.2 Project Management Group (PMG)

The study is supervised by its Project management Group (PMG). This consists of the grant holders and representatives from the Study Office. Observers may be invited to attend at the discretion of the PMG. We will meet/teleconference every six months on average.

The research team has the expertise to cover the clinical and surgical aspects of the research.

10.3 Trial Steering Committee (TSC)

The study is overseen by a Trial Steering Committee (TSC). The membership comprises the four independent members (including the Chairman), and the CI (or a deputy). The other grant holders, a representative from the sponsoring institution and the funders (the HTA) may also attend, as may

other members of the TISU study office or members of other professional bodies at the invitation of the Chair.

10.4 Data Monitoring Committee (DMC)

The DMC is made up of members listed at the start of this protocol. The DMC will initially meet to agree its terms of reference and other procedures. CHaRT has adopted the DAMOCLES Charter for DMCs and suggests to the independent DMC members that they adopt the Terms of reference contained within.

The committee will meet regularly to monitor the unblinded trial data and serious adverse events and make recommendations as to any modifications that are required to be made to the protocol or the termination of all or part of the trial.

These interim reports will be supplied in strict confidence to the DMC. Frequency of meetings will depend on the judgement of the chairman and other independent members of the DMC. The reports to the DMC will be held in the strictest confidence by its members and securely archived.

10.5 Local organisation in sites

Lead Urologist (Local Principal Investigator)

Each collaborating centre will identify a Lead Urologist who will be the point of contact for that centre. The responsibilities of this person will be to:

- establish the study locally (for example, by getting agreement from clinical colleagues; facilitate local regulatory approvals; identify, appoint and train a local Research Nurse; and inform all relevant local staff about the study (eg other consultant urologists, junior medical staff, secretaries, ward staff))
- take responsibility for clinical aspects of the study locally (for example if any particular concerns occur)
- identify and/or support colleagues to identify potential participants
- notify the Study Office of any unexpected clinical events which might be related to study participation
- provide support, training and supervision for the local Research Nurse(s)
- represent the centre at the collaborators' meetings.

Local Research Nurse

Each collaborating centre will appoint a local Research Nurse to organise the day to day recruitment of participants to the study. The responsibilities of this person will be to:

- keep regular contact with the local Lead Urologist, with notification of any problem or unexpected development
- maintain regular contact with the TISU Study Office
- keep local staff informed of progress in the study
- identify any eligible patients at clinics or on the ward while they are in hospital; explain the study and the potential for participation in TISU if they are eligible
- obtain patient's written consent
- keep a log of whether patients are recruited or not (with reasons for non-participation)
- collect baseline data describing the participant, log this information in the web-based TISU database and send paper copies to the Study Office along with the original signed consent forms
- use this information to randomise the participant
- ensure treatment and post treatment data are collected and recorded in the web-based TISU database, and send paper copies (as requested) to the Study Office
- file relevant study documentation (eg consent forms) in the participant's medical records
- organise alternative recruiters in case of holiday or absence
- represent the centre at the collaborators' meetings.

11 RESEARCH GOVERNANCE, SPONSORSHIP AND DATA PROTECTION

11.1Research Governance

The trial will be run under the auspices of CHaRT based at HSRU, University of Aberdeen. This will ensure compliance with Research Governance, and provide centralised trial administration, database support and economic and statistical analyses. CHaRT is a registered Clinical Trials Unit with particular expertise in running multicentre RCTs of complex and surgical interventions.

The CI will ensure, through the TSC, that adequate systems are in place for monitoring the quality of the study (compliance with GCP) and appropriate expedited and routine reports, to a level appropriate to the risk assessment of the study.

11.2 Data protection

Data collected during the course of the research will be kept strictly confidential and accessed only by members of the trial team. Participant's details will be stored on a secure database under the guidelines of the 1988 Data Protection Act and regular checks and monitoring are in place to ensure compliance. Data are stored securely in accordance with the Act and archived to a secure data storage facility. The senior IT manager (in collaboration with the Chief Investigator) will manage access rights to the data set.. Participants will be allocated an individual specific trial number and their details will be anonymised on the secure database. We anticipate that anonymised trial data may be shared with other researchers to enable international prospective meta-analyses. To comply with the 5th Principle of the Data Protection Act 1998, personal data will not be kept for longer than is required for the purpose for which it has been acquired.

11.3 Sponsorship

The University of Aberdeen and NHS Grampian are the co-sponsors for the trial.

12. ETHICAL ISSUES AND ARRANGEMENTS

The North of Scotland Research Ethics Committee 2 has reviewed this study. The study will be conducted according to the principles of good practice provided by Research Governance Guidelines. We believe this study does not pose any specific risks to individual participants beyond those of any ureteric stone treatment, nor does it raise any extraordinary ethical issues. Annual progress reports and a final report at the conclusion of the trial will be submitted to North of Scotland REC within the timelines defined in the regulations.

13. QUALITY ASSURANCE

The trial will be monitored to ensure that the study is being conducted as per protocol, adhering to Research Governance, GCP and the appropriate legislation. The approach to, and extent of, monitoring (specifying both central and on-site monitoring) will be specified in a trial monitoring plan which is usually initially determined by a risk assessment, undertaken prior to start of trial.

14. FINANCE AND INSURANCE

The trial is funded by a grant awarded by the NIHR Health Technology Assessment programme.

The necessary trial insurance is provided by the University of Aberdeen.

15. DATA HANDLING, RECORD KEEPING AND ARCHIVING

Clinical data will be entered into the database by the local investigator and/or research nurse working in each hospital site, together with data from questionnaires completed at clinic. Questionnaires returned by post to the trial office will be entered there. Staff in the trial office will work closely with local research nurses to ensure that the data are as complete and accurate as possible. Extensive range and consistency checks will further enhance the quality of the data.

The co-sponsors are responsible for ensuring that trial data is archived appropriately. Essential data shall be retained for a period of at least 10 years following close of study.

16. SATELLITE STUDIES

It is recognised that the value of the study may be enhanced by smaller ancillary studies of specific aspects. Plans for these will be discussed in advanced with the Project Management Group. REC approval will be sought for any new proposal, if appropriate.

17. AUTHORSHIP AND PUBLICATION

All RCTs conducted by CHaRT have a commitment to publish the findings of the research. At a minimum this trial will have a results paper published in a peer-reviewed medical/scientific journal.

If all grant-holders and researcher staff fulfil authorship rules, group authorship will be used under the collective title of ‘the TISU Trial Group’. If one or more individuals have made a significant contribution above and beyond other group members but where all group members fulfil authorship rules, authorship will be attributed to the named individual(s) and the TISU Trial Group.

For reports which specifically arise from the trial but where all members do not fulfil authorship rules (for example, specialist sub-study publications), authorship should be attributed to the named individual(s) for the TISU Trial Group.

To safeguard the integrity of the main trial, reports of explanatory or satellite studies will not be submitted for publication without prior agreement from the Project Management Group.

We intend to maintain interest in the study by publication of TISU newsletters at intervals for staff and collaborators. Once the main report has been published, a lay summary of the findings will be sent in a final TISU Newsletter to all involved in the trial including trial participants.

Further details on the publication policy can be found in Appendix 2.

REFERENCE LIST

1 Menon M, Parulkar BG, Drach GW. Urinary lithiasis: etiology, diagnosis and medical management. In: Walsh PC, Retnik AB, Vaughan ED, Wein AJ, editors. Campbell's Urology. 7th ed. Philadelphia: WB Saunders Company; 1998. p. 2659-2752.

2 Bihl G, Meyers A. Recurrent renal stone disease-advances in pathogenesis and clinical management. Lancet 2001 Aug 25;358(9282):651-656.

3 Wilkinson H. Clinical investigation and management of patients with renal stones. Ann Clin Biochem 2001 May;38(Pt 3):180-187.

4 Stamatelou KK, Francis ME, Jones CA, Nyberg LM, Curhan GC. Time trends in reported prevalence of kidney stones in the United States: 1976-1994. Kidney Int 2003 May;63(5):1817-1823.

5 Hospital Episode Statistics . Inpatient data 2011-2012 [spreadsheet on the Internet]. 2012; Available at: http://www.hesonline.nhs.uk/Ease/servlet/ContentServer?siteID=1937&categoryID=192, [accessed November 2012].

6 Preminger GM. Tiselius HG. Assimos DG. Alken P. Buck AC. Gallucci M. Knoll T. Lingeman JE. Nakada SY. Pearle MS. Sarica K. Turk C. Wolf JS Jr. American Urological Association Education and Research, Inc. European Association of Urology. 2007 Guideline for the management of ureteral calculi. Eur Urol 2007 Dec;52(6):1610-1631.

7 Gerber GS, Acharya SS. Management of Ureteral Calculi. Journal of Endourology 2010;24(6):953-954.

8 Nabi G, Downey P, Keeley F, Watson G, McClinton S. Extra-corporeal shock wave lithotripsy (ESWL) versus ureteroscopic management for ureteric calculi. Cochrane Database Syst Rev 2007 Jan 24;(1)(1):CD006029.

9 Aboumarzouk OM, Kata SG, Keeley FX, McClinton S, Nabi G. Extracorporeal shock wave lithotripsy (ESWL) versus ureteroscopic management for ureteric calculi. Cochrane Database of Syst Rev 2012, Issue 5. Art. No: CD006029. DOI: 10.1002/14651858.CD006029.pub4.

10 Downie WW, Leatham PA, Rhind VM, Wright V, Branco JA, Anderson JA. Studies with pain rating scales. Ann Rheum Dis 1978 Aug;37(4):378-381.

11 Kind P, Hardman G, Macran S. Population norms for EQ-5D. Centre for Health Economics Discussion Paper 172. York: University of York; 1999.

Appendix 1

Revised Project Timetable and Milestones

The study duration is 66 months including an internal feasibility phase:

Months: 1-5: Study initiation, NHS approvals;
Months: 6-18: Internal feasibility; staggered site start up; recruit participants;
Months: 18-53: Continue to recruit patients;
Months: 11-60: Patient follow up completed;
Months: 61--66: Close down, analysis, report writing.

***The Gantt chart of trial progress for further information*)**
Figure 4: Gantt chart of trial progress

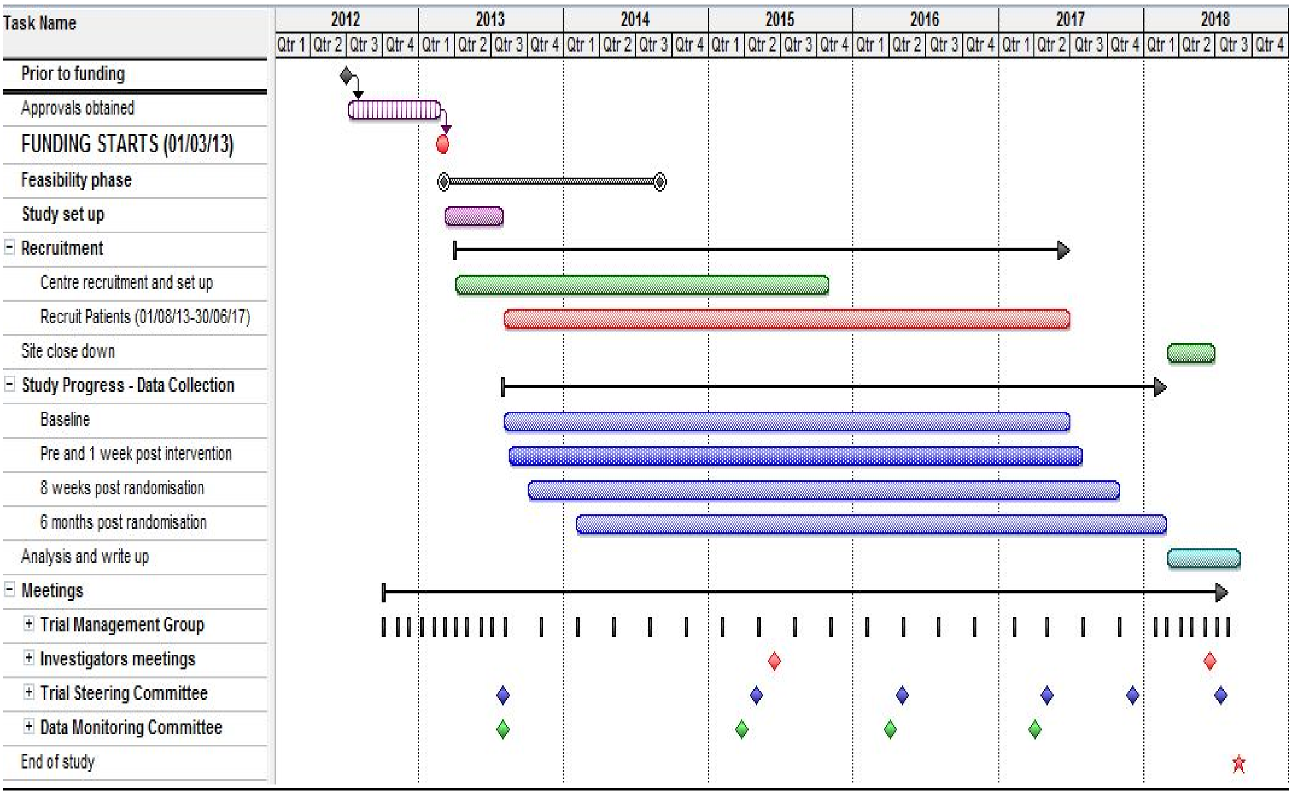

Appendix 2: Authorship Policy

1. PRINCIPLES OF AUTHORSHIP

The following principles of authorship have been derived from editorial publications from leading journals (see references) and are in accordance with the rules of the international Committee of Medical Journal Editors.

a. Group authorship

Group authorship will be appropriate for some publications, such as main reports. This will apply when the intellectual work underpinning a publication 'has been carried out by a group, and no one person can be identified as having substantially greater responsibility for its contents than others'.[1] In such cases the authorship will be presented by the collective title - The TISU Trial Group - and the article should carry a footnote of the names of the people (and their institutions) represented by the corporate title. In some situations one or more authors may take responsibility for drafting the paper but all group members qualify as members; in this case, this should be recognised using the by-line 'Jane Doe *and* the Trial Group'.[2] Group authorship may also be appropriate for publications where one or more authors take responsibility for a group, in which case the other group members are not authors but may be listed in the acknowledgement (the by-line would read 'Jane Doe *for* the Trial Group').[2]

b. Individual authorship

Other papers, such as describing satellite studies, will have individual authorship. In order to qualify for authorship an individual must fulfil the following criteria[1]:

i. each author should have participated sufficiently in the work represented by the article to take public responsibility for the content.

ii. participation must include three steps:

- conception or design of the work represented by the article OR analysis and interpretation of the data OR both; AND
- drafting the article or revising it for critically important content; AND
- final approval of the version to be published.

Participation solely in the collection of data is insufficient by itself. Those contributors who do not justify authorship may be acknowledged and their contribution described.[1]

c. Determining authorship

Tentative decisions on authorship should be made as soon as possible[1]. These should be justified to, and agreed by, the Project Management Group. Any difficulties or disagreements will be resolved by the Steering Committee.

2. AUTHORSHIP FOR PUBLICATION ARISING FROM TISU

a. Operationalising authorship rules

We envisage two types of report (including conference presentations) arising from the TISU trial and its associated projects:

i. *Reports of work arising from the main* TISU trial

If all grant-holders and research staff fulfil authorship rules, group authorship should be used under the collective title of 'The TISU Trial Group'; if one or more individuals have made a significant contribution above and beyond other group members but where all group members fulfil authorship rules, authorship will be attributed to 'Jane Doe and the TISU Trial Group'.

ii. *Reports of satellite studies and subsidiary projects*

Authorship should be guided by the authorship rules outlined in Section 1 above. Grant-holders and research staff not directly associated with the specific project should only be included as authors if they fulfil the authorship rules. Grant-holders and research staff who have made a contribution to the project but do not fulfil authorship rules should be recognised in the Acknowledgement section. The

role of the TISU Trial Group in the development and support of the project should be recognised in the Acknowledgement section. The lead researcher should be responsible for ratifying authorship with the Project Management Group.

For reports which specifically arise from the TISU trial but where all members do not fulfil authorship rules (for example, specialist sub-study publications), authorship should be attributed to 'Jane Doe for the TISU Trial Group'. If individual members of the group are dissatisfied by a decision, they can appeal to the Project Management Group for reconciliation. If this cannot be achieved, the matter should be referred to the TSC.

b. Quality assurance

Ensuring quality assurance is essential to the good name of the trial group. For reports of individual projects, internal peer review among members of the PMG is a requirement prior to submission of papers. All reports of work arising from TISU trial including conference abstracts should be peer reviewed by the PMG.

The internal peer review for reports of work arising from the TISU project is mandatory and submission may be delayed or vetoed if there are serious concerns about the scientific quality of the report. The Project Management Group will be responsible for decisions about submission following internal peer review. If individual members of the group are dissatisfied by decisions, the matter may be referred to the TSC.

The PMG undertakes to respond to submission of articles for peer review at the PMG meeting following submission (assuming the report is submitted to the trial secretariat in Aberdeen at least two weeks prior to the meeting).

3. REFERENCES

1. Huth EJ (1986). Guidelines on authorship of medical papers. *Annals of Internal Medicine*, **104**, 269-274.
2. Glass RM (1992). New information for authors and readers. Group authorship, acknowledgements and rejected manuscripts. *Journal of the American Medical Association*, **268**, 99.
